# Gestational Diabetes Mellitus Is Associated with Reduced Dynamics of Gut Microbiota during the First Half of Pregnancy

**DOI:** 10.1128/mSystems.00109-20

**Published:** 2020-03-24

**Authors:** Wei Zheng, Qian Xu, Wenyu Huang, Qi Yan, Yating Chen, Li Zhang, Zhihong Tian, Ting Liu, Xianxian Yuan, Cheng Liu, Jinying Luo, Cuimei Guo, Wei Song, Lirui Zhang, Xin Liang, Huanlong Qin, Guanghui Li

**Affiliations:** a Division of Endocrinology and Metabolism, Department of Obstetrics, Beijing Obstetrics and Gynecology Hospital, Capital Medical University, Beijing, China; b Realbio Genomics Institute, Shanghai, China; c Shanghai Tenth People’s Hospital, Tongji University School of Medicine, Shanghai, China; d Division of Endocrinology, Metabolism and Molecular Medicine, Northwestern University Feinberg School of Medicine, Chicago, Illinois, USA; e Department of Obstetrics, Fujian Provincial Maternity and Children’s Hospital, Affiliated Hospital of Fujian Medical University, Fuzhou, China; Institute for Systems Biology

**Keywords:** gestational diabetes mellitus, GDM, gut microbiota, first trimester, T1, second trimester, T2

## Abstract

Women with gestational diabetes mellitus (GDM) have different gut microbiota in late pregnancy compared to women without GDM. It remains unclear whether alterations of gut microbiota can be identified prior to the diagnosis of GDM. This study characterized dynamic changes of gut microbiota from the first trimester (T1) to the second trimester (T2) and evaluated their relationship with later development of GDM. Compared with the control group (*n* = 103), the GDM group (*n* = 31) exhibited distinct dynamics of gut microbiota, evidenced by taxonomic, functional, and structural shifts from T1 to T2. Linear discriminant analysis (LDA) revealed that there were 10 taxa in T1 and 7 in T2 that differed in relative abundance between the GDM and control groups, including a consistent decrease in the levels of *Coprococcus* and *Streptococcus* in the GDM group. While the normoglycemic women exhibited substantial variations of gut microbiota from T1 to T2, their GDM-developing counterparts exhibited clearly reduced inter-time point shifts, as corroborated by the results of Wilcoxon signed-rank test and balance tree analysis. Moreover, cooccurrence network analysis revealed that the interbacterial interactions in the GDM group were minimal compared with those in the control group. In conclusion, lower numbers of dynamic changes in gut microbiota in the first half of pregnancy are associated with the development of GDM.

**IMPORTANCE** GDM is one of the most common metabolic disorders during pregnancy and is associated with adverse short-term and long-term maternal and fetal outcomes. The aim of this study was to examine the connection between dynamic variations in gut microbiota and development of GDM. Whereas shifts in gut microbiota composition and function have been previously reported to be associated with GDM, very little is known regarding the early microbial changes that occur before the diagnosis of GDM. This study demonstrated that the dynamics in gut microbiota during the first half of pregnancy differed significantly between GDM and normoglycemic women. Our findings suggested that gut microbiota may potentially serve as an early biomarker for GDM.

## Abstract (precis)

GDM is one of the most common metabolic disorders during pregnancy and is associated with adverse short-term and long-term maternal and fetal outcomes. The aim of this study was to examine the connection between dynamic variations in gut microbiota and development of GDM. Whereas shifts in gut microbiota composition and function have been previously reported to be associated with GDM, very little is known regarding the early microbial changes that occur before the diagnosis of GDM. This study demonstrated that the dynamics in gut microbiota during the first half of pregnancy differed significantly between GDM and normoglycemic women. Our findings suggested that gut microbiota may potentially serve as an early biomarker for GDM.

## INTRODUCTION

Gestational diabetes mellitus (GDM) affects up to 25.1% of pregnancies worldwide (1). Importantly, the disorder is associated with a variety of adverse maternal and neonatal outcomes, including fetal macrosomia, preeclampsia, and cesarean delivery (2, 3). GDM also has long-term maternal metabolic ramifications, particularly an increased risk of diabetes, hypertension, dyslipidemia, and coronary heart disease (4, 5). Therefore, early recognition of GDM is critical in preventing these complications.

The pathogenesis of GDM is not well understood. Recent studies have demonstrated an important role of gut microbiota in the pathogenesis of a variety of metabolic disorders, including diabetes (6–8), cardiovascular disease (9), and obesity (10), as well as in the treatment of these disorders (11). Shifts in gut microbiota at late pregnancy have been shown in women with GDM (12–14). Wang et al. (13) reported a strong association between certain discriminatory bacteria and glucose levels in late pregnancy. Another study demonstrated that dysbiosis (perturbation of healthy states) in gut microbiota was found in GDM women during late pregnancy and 8 months postpartum which resembled the aberrant microbiota composition in patients with type 2 diabetes (14). These findings indicated that shifts in gut microbiota may be associated with GDM. Additionally, prominent changes in the enteric microbial community through different trimesters likely represent an intrinsic property of normal pregnancy (12). These alterations in gut microbiota occur along with metabolic changes during normal pregnancy, including an increase in endogenous glucose production and a decrease in insulin sensitivity by late gestation (15).

Although changes in gut microbiota are implicated in insulin sensitivity (16), their role in the pathogenesis of GDM remains poorly understood. In addition, since GDM is typically diagnosed around gestational weeks 24 to 28 and patients may have received behavioral, nutritional, and/or pharmacological intervention afterward, the discovery of altered gut microbiota composition during late pregnancy and postpartum may be confounded by these interventions (17). Therefore, the aim of this study was to investigate the association between variations in gut microbiota during the 1st and 2nd trimesters and development of GDM.

## RESULTS

### Clinical characteristics of the study subjects.

Among the participants with singleton pregnancies, 31 developed GDM whereas 103 were normoglycemic between 24 and 28 weeks of gestation. The GDM and control groups were similar in age, gravidity, and parity (Table 1). Women in the GDM group had a higher prepregnancy body mass index (BMI) and higher levels of total cholesterol (TC) and total triglyceride (TG) than the normoglycemic participants during early pregnancy, which was consistent with previous reports (18, 19).

**TABLE 1:** Clinical characteristics of women with and without GDM^*a*^

Characteristic	Values		*P* value
	Women with GDM (*n* = 31)	Normoglycemic women (*n* = 103)	
General information			
Age (yr), mean ± SD	32.58 ± 4.1	31.79 ± 3.70	0.36
Gravidity (first pregnancy), *n* (%)	14 (45.16)	53 (51.46)	0.54
Multipara, *n* (%)	9 (29.03)	31 (30.01)	0.91
History of adverse pregnancy outcomes, *n* (%)	3 (9.68)	14 (13.59)	0.57
Polycystic ovary syndrome, *n* (%)	1 (3.23)	9 (8.74)	0.31
Smoking, *n* (%)	2 (6.67)	4 (3.96)	0.53
Family history of diabetes, *n* (%)	8 (25.81)	13 (12.62)	0.08
Anthropometric measurements			
Ht (cm), median (IQR)	163.0 (158.0–165.0)	162.0 (160.0–168.0)	0.67
Wt (kg) prepregnancy, median (IQR)	60.0 (53.0–67.0)	56.83 (50.00–63.00)	0.049
BMI (kg/m^2^) prepregnancy, mean ± SD	22.57 ± 2.85	21.32 ± 3.00	0.04
Overweight/obese (BMI≥25 kg/m^2^) prepregnancy, *n* (%)	9 (29.03)	13 (12.56)	0.03
Gestational weight gain (kg) at OGTT, mean ± SD	6.89 ± 3.03	6.91 ± 2.97	0.85
Biochemical indicator in first trimester			
Fasting blood glucose (mmol/liter), median (IQR)	4.75 (4.53–5.01)	4.67 (4.41–4.92)	0.21
Blood lipid level (mmol/liter), median (IQR)			
TC	4.41 (3.96–4.94)	4.10 (3.66–4.52)	0.04
TG	1.29 (0.92–1.76)	1.00 (0.77–1.32)	0.02
HDL-C	1.55 (1.29–1.70)	1.43 (1.23–1.64)	0.19
LDL-C	2.16 (1.75–2.66)	2.14 (1.72–2.38)	0.17
Gestational wk at examination, median (IQR)	8.0 (7.0–9.0)	9.0 (8.0–10.0)	0.31
Biochemical indicator in second trimester			
Blood glucose level (mmol/liter) at OGTT, median (IQR)			
Fasted	4.84 (4.53–5.20)	4.46 (4.26–4.66)	<0.0001
1 h	10.36 (9.34–10.88)	7.33 (6.06–8.39)	<0.0001
2 h	8.21 (7.42–8.90)	6.14 (5.40–7.07)	<0.0001
Blood lipid level (mmol/liter), median (IQR)			
TC	5.68 (5.29–6.19)	5.90 (5.24–6.48)	0.55
TG	2.34 (1.89–2.98)	2.06 (1.61–2.85)	0.55
HDL-C	1.77 (1.67–1.92)	1.88 (1.57–2.08)	0.67
LDL-C	2.87 (2.50–3.38)	3.09 (2.59–3.49)	0.48
Gestational wk at examination, median (IQR)	24.0 (24.0–25.0)	24.0 (24.0–25.0)	0.42

a GDM, gestational diabetes mellitus; IQR, interquartile range; BMI, body mass index; OGTT, oral glucose tolerance test; TC, total cholesterol; TG, triglycerides; HDL-C, high-density lipoprotein-cholesterol; LDL-C, low-density lipoprotein-cholesterol.

### Composition and structure of the intestinal bacterial community.

16S rRNA gene amplicon sequencing for the stool samples generated 9,467,274 high-quality reads, resulting in an average yield of 35,325.65 clean reads per sample. At a 97% similarity threshold, the clean reads were clustered using USEARCH (Robert C. Edgar) to produce 1,390 operational taxonomic units (OTUs), which were subsequently assigned to the RDP database (release 11) to generate taxonomic annotations. Eventually, 1,296 OTUs were taxonomically annotated (confidence coefficient value, >0.8), including 594 OTUs at the genus level.

Examination of the microbial structure revealed no differences between the GDM and control groups in both the first trimester (T1) and the second trimester (T2) (see Fig. S1A and B in the supplemental material). Between the two time points, there were significant changes (*P* = 0.041) in the alpha diversity of the GDM group (Fig. S1A) and a shift in the results of principal-coordinate analysis (PCoA) in the control group (Fig. S1C). Taxonomically, *Firmicutes* and *Bacteroidetes* were the dominant phyla, followed by *Proteobacteria* and *Actinobacteria* (Fig. S2). In both control and GDM groups, there was a comparable increase in the *Firmicutes*/*Bacteroidetes* ratio (F/B ratio) from T1 to T2 (0.852 in T1 to 1.10 in T2 in the control group and 0.892 in T1 to 1.10 in T2 in the GDM group; *P* < 0.05). At the genus level, the top taxa were *Bacteroides*, *Prevotella*, *Faecalibacterium*, and *Roseburia* and the genus-level compositions of many taxa were comparable between the GDM and control groups (Fig. S2). Overall, our data indicated that the control and GDM groups shared considerable similarities in gut microbiota and that the microbiota structure in either group was relatively stable between T1 and T2.

10.1128/mSystems.00109-20.1 FIG S1 Diversity (A) and dissimilarity in microbial composition principal coordinates analysis (PCoA) (B) between the GDM and control groups in both T1 and T2. Paired samples were used for inter-time point comparison between T1 and T2 within the GDM (C) or normoglycemic group (D). Green boxes represent women with GDM in T1; brown boxes represent women with GDM in T2; blue boxes represent normoglycemic women in T1; orange boxes represent normoglycemic women in T1. Download FIG S1, PDF file, 1.6 MB. Copyright © 2020 Zheng et al. 2020 Zheng et al. This content is distributed under the terms of the Creative Commons Attribution 4.0 International license.

10.1128/mSystems.00109-20.2 FIG S2 Microbial composition of each group (A and C) and individual participants (B and D) shown at phylum and genus levels in T1 and T2. Download FIG S2, PDF file, 1.1 MB. Copyright © 2020 Zheng et al. 2020 Zheng et al. This content is distributed under the terms of the Creative Commons Attribution 4.0 International license.

### Taxonomic biomarkers of GDM.

**FIG 1:**
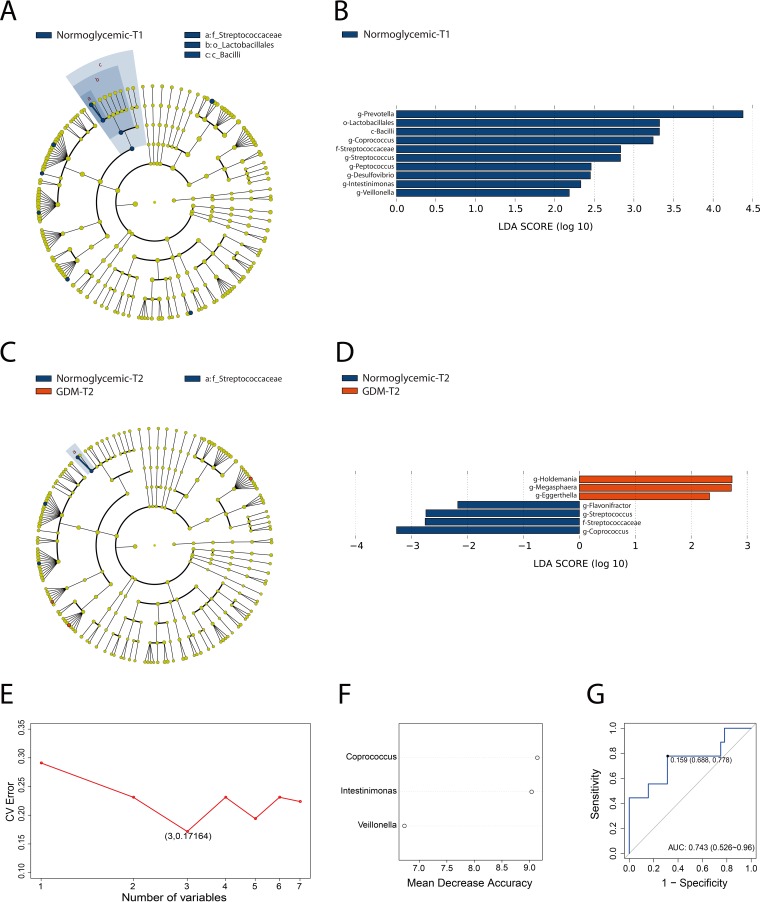
Gut microbial biomarkers of GDM in the first and second trimesters. Cladogram (A and C) and scores (B and D) of taxonomic biomarkers between women with GDM and control group identified by linear discriminant analysis (LDA). Red bars indicate the bacterial taxa with greater relative abundance in the GDM group; blue bars indicate the bacterial taxa with greater relative abundance in the control group. (E to G) A predictive model of GDM onset based on abundance profile in T1 derived from random forest analysis. (E) Relationship between the numbers of taxa included in the random forest model and the corresponding predictive performance (estimated by 10-fold cross-validation). CV, coefficient of variation. (F) The three genera in the predictive model. (G) The receiver operating characteristic (ROC) curve for predicting GDM onset generated by random forest; the plots shown in the panels represent the corresponding optimal thresholds.

Despite the overall similarity of microbial structure between the GDM and control groups, linear discriminant analysis (LDA) identified multiple different taxa between the two groups at both early gestation and midgestation (Fig. 1). In T1, 10 taxa were found to have high relative abundance in the normoglycemic group compared to none in the GDM group. At the genus level, the differential taxa were *Prevotella*, *Coprococcus*, *Streptococcus*, *Peptococcus*, *Desulfovibrio*, *Intestinimonas*, and *Veillonella*. Furthermore, the parent taxa of *Streptococcus* (i.e., the family *Streptococcaceae*, order *Lactobacillales*, and class *Bacilli*) differed significantly between the two groups. In T2, 7 taxa were found to be significantly different between the two groups. While *Holdemania*, *Megasphaera*, and *Eggerthella* displayed higher relative abundance in the GDM group, *Flavonifractor*, *Streptococcus* (along with its parent family *Streptococcaceae*), and *Coprococcus* were more abundant in the control group. Notably, *Coprococcus*, a genus of butyrate-producing bacteria, and *Streptococcus* (and its parent family *Streptococcaceae*), a genus of lactate-producing bacteria, were less abundant in the GDM group in both T1 and T2. In addition, the proportions of *Megasphaera* and *Eggerthella* in the GDM group increased, which was consistent with a previous report (20). To investigate whether the gut microbiota in T1 can potentially be used as early biomarkers of GDM, random forest analysis was performed to generate a 3-component panel of *Coprococcus*, *Intestinimonas*, and *Veillonella* that displayed a moderately good performance (area under the concentration-time curve [AUC] of 0.743 mg · h/liter) in predicting GDM diagnosis for this training data set (Fig. 1E and F).

### Dynamics of bacterial composition and function from early to midgestation.

**FIG 2:**
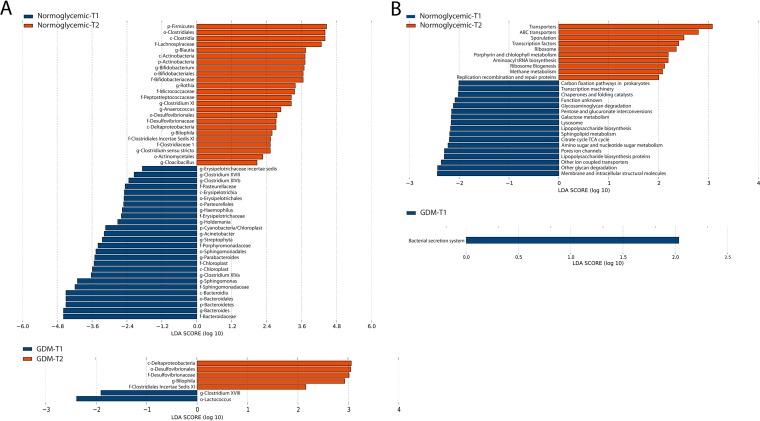
Dynamic changes of gut microbiota from T1 to T2. (A and B) Differential taxa (A) and functions (B) generated by LDA. Red bars indicate bacterial taxa and functions with significantly greater relative abundance in T2; blue bars indicate bacterial taxa and functions with greater relative abundance in T1. TCA cycle, tricarboxylic acid cycle.

We next investigated the inter-time point dynamics of gut microbiota from T1 to T2 in the control and GDM groups. LDA revealed prominent taxonomic differences between the first and second trimesters in the control group, with 49 taxa exhibiting different relative abundances between early gestation and midgestation (*P* < 0.05, LDA > 1.5) (Fig. 2A). Specifically, 25 taxa showed greater relative proportions in T1, as did 24 taxa in T2. At the phylum level, there was an increased relative abundance of *Firmicutes* and a decreased relative abundance of *Bacteroides* in T2 (Fig. S2). At the genus level, *Bacteroides*, *Sphingomonas*, *Parabacteroides*, *Streptophyta*, Acinetobacter, *Holdemania*, *Haemophilus*, *Clostridium* XVIII, *Clostridium* XIVb, and Erysipelotrichaceae incertae *sedis* were more abundant in T1, while *Blautia*, *Bifidobacterium*, *Rothia*, *Clostridium* XI, *Anaerococcus*, *Bilophila*, and *Clostridium sensu stricto* displayed greater relative abundances in T2. In comparison, only 7 taxa were differentially abundant between T1 and T2 in the GDM group. At the genus level, 3 taxa registered inter-time point changes, including *Bilophila* being more abundant and *Clostridium* XVIII and *Lactococcus* being less abundant in T2. Interestingly, despite significant differences in the gut microbiota dynamics between GDM and the normoglycemic subjects, 5 taxa showed consistent inter-time point shifts in both groups. Specifically, *Clostridium* XVIII had a greater relative abundance in both groups in T1, as did the genus *Bilophila* and class Deltaproteobacteria (along with its parent order *Desulfovibrionales* and family *Desulfovibrionaceae*) in T2. Wilcoxon signed-rank test was employed to further validate the aforementioned taxonomic differences between the early gestational and midgestational periods. This revealed 14 genera (e.g., *Prevotella*, *Blautia*, *Bifidobacterium*, *Parabacteroides*, and *Bacteroides*) with differential relative abundances between T1 and T2 in the control group but only 1 genus of *Bilophila* that differed between T1 and T2 in the GDM group (Fig. S3A and B). Importantly, there was considerable concordance between the differential genera identified by LDA and Wilcoxon signed-rank test (Fig. 2A; see also Fig. S3A and B). Of note, the aberrant microbiota dynamics in the GDM group were not a result of particular outlier samples (Fig. S1D; see also Fig. S4). Despite the substantial intergroup and inter-time point microbiota shifts, Spearman analysis did not detect any significant associations between microbial taxa and glucolipid measures, including fasting plasma glucose, lipid profiles, homeostatic model assessment-insulin resistance (HOMA-IR) score, and HOMA β-cell index.

10.1128/mSystems.00109-20.3 FIG S3 Microbial features that displayed differential relative abundance between T1 and T2 based on Wilcoxon signed-rank test, including differential genera in the control group (A) and GDM group (B) as well as PICRUSt-predicted functions in the control group (C). There was no statistically significant difference between the T1 data and the T2 data in any of the PICRUSt-predicted functions in the GDM group. Download FIG S3, PDF file, 0.8 MB. Copyright © 2020 Zheng et al. 2020 Zheng et al. This content is distributed under the terms of the Creative Commons Attribution 4.0 International license.

10.1128/mSystems.00109-20.4 FIG S4 The relative abundance data of the differential inter-time point taxa in individual participants of the control (A) and GDM (B) groups. Download FIG S4, PDF file, 3.1 MB. Copyright © 2020 Zheng et al. 2020 Zheng et al. This content is distributed under the terms of the Creative Commons Attribution 4.0 International license.

We next examined the changes in the dynamics of microbial functions from T1 to T2. Similarly to our observations in taxonomic composition, PICRUSt-based prediction and statistical analyses indicated that substantial functional differences between T1 and T2 were apparent in the control group but absent in the GDM group (Fig. 2B; see also Fig. S3C). Overall, compared with the normoglycemic subjects, women who developed GDM exhibited relatively stable gut microbiota in both taxonomy and function from early to midgestation, suggesting a connection between aberrant host metabolic functions and diminished enteric microbial dynamics.

### Balance tree analysis of gut microbiota from the first to second trimesters.

**FIG 3:**
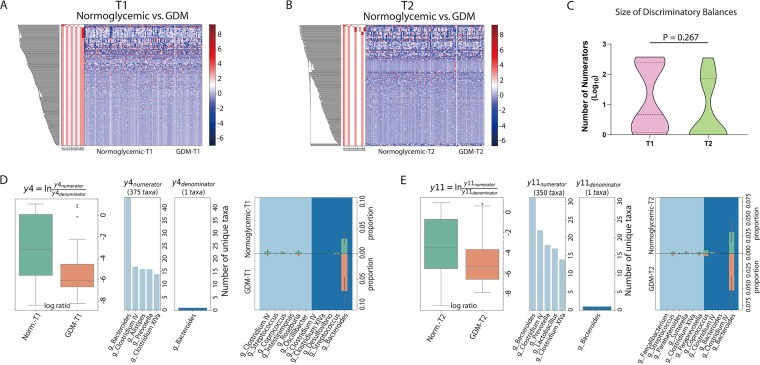
(A and B) Balance tree analysis of the T1 (A) and T2 (B) intergroup microbiota differences. (C) Taxon number comparison of the intergroup discriminatory balances (FDR < 0.1) between T1 and T2. (D and E) Representative large intergroup discriminatory balance in T1 (D) or T2 (E). The presented traits of each branch include log ratio, taxonomic composition, and T1-T2 proportions of individual taxa (D and E). Norm., normoglycemic.

Because the change in the proportion of one microbe may influence those of others within a microbial community, balance tree analysis has been proposed to elucidate the interdependent alterations of microbial clusters (21). A comparative analysis using comparisons between the GDM and control groups in either trimester generated 12 or 11 discriminatory balances (false-discovery rate [FDR] < 0.1). Of note, there was a wide variation in the size of these signature clusters. We introduced here a threshold of 20 taxa to classify large (≥20 OTUs) and small (<20 OTUs) discriminatory balances; a large balance typically encompassed over 100 OTUs, whereas small ones mostly had fewer than 10 OTUs (Fig. S4 and S5; see also Table S3 and Table S4 in the supplemental material). In either T1 or T2, there were 5 or 4 large balances distinguishing the GDM and control groups (Fig. 3A and B; see also Fig. S5 and Table S3); the average taxon numbers of intergroup discriminatory balances were comparable between the two time points (Fig. 3C). There were considerable variations in the taxonomic composition of the large balances in both the numerator clusters and the denominator clusters (Table S4). However, the balance tree analysis results for Y4 of T1 and Y11 of T2 (i.e., two individual large balances at each time point) showed highly concordant taxonomic compositions, including identical denominators (*Bacteroides*, OTU_7) (Table S4). Importantly, the two balances exhibited similar shifts not only as a whole but also in individual constituent taxa, e.g., *Bacteroides*, *Clostridium* IV, *Streptococcus*, *Coprococcus*, and *Clostridium* XIVa (Fig. 3D and E). Hence, despite substantial differences in the microbiota shifts in the GDM-developing women at the two time points, a major trend (i.e., represented by the balances Y4 of T1 and Y11 of T2) was conserved from early pregnancy to midpregnancy.

10.1128/mSystems.00109-20.5 FIG S5 Examination of microbial differences between T1 and T2 in the control (A) and GDM (B) groups, respectively, based on balance tree analysis. Download FIG S5, PDF file, 2.7 MB. Copyright © 2020 Zheng et al. 2020 Zheng et al. This content is distributed under the terms of the Creative Commons Attribution 4.0 International license.

**FIG 4:**
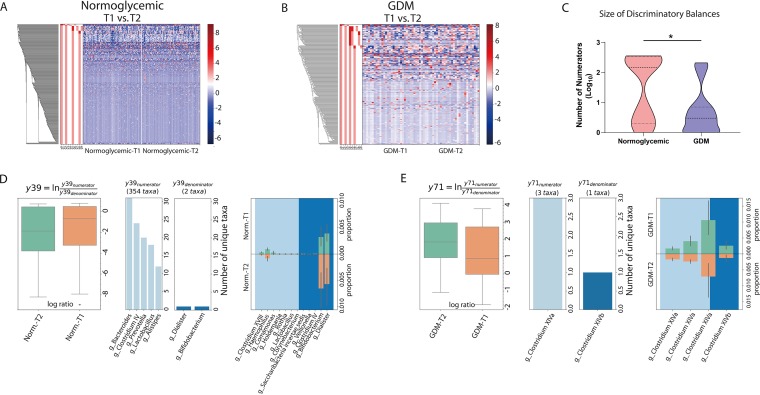
(A and B) Balance tree analysis of the inter-time point microbiota differences in the normoglycemic (A) and GDM (B) groups. (C) Taxon number comparison of the inter-time point discriminatory balances (FDR < 0.1) between the control and GDM groups. (D and E) Representative large inter-time point discriminatory balance in the control (D) or GDM (E) group. The traits of each branch presented in panels D and E include log ratio, taxonomic composition, and T1-T2 proportions of individual taxa. Norm., normoglycemic.

We next employed this method to investigate inter-time point changes in the participants. This analysis revealed apparent shifts in both the GDM and control groups, evidenced by around 20 discriminatory balances (FDR < 0.1) in either group of participants (Fig. 4A and B; see also Fig. S5 and Table S3). Notably, the average taxon numbers of discriminatory balances in the GDM group were significantly lower than those of the control group (*P* < 0.05; Wilcoxon signed-rank test; Fig. 4C), consistent with the fact that slightly over half (10/18) of the discriminatory balances in the control group were large, whereas the majority (19/23) of such balances in the GDM group were small (Fig. 4A and B; see also Fig. S6 and Table S3). These results further underscored the diminished microbiota shifts seen between T1 and T2 in the GDM-developing participants.

10.1128/mSystems.00109-20.6 FIG S6 Examination of microbial differences between GDM and normoglycemic women in T1 (A) and T2 (B), respectively, based on balance tree analysis. Download FIG S6, PDF file, 1.1 MB. Copyright © 2020 Zheng et al. 2020 Zheng et al. This content is distributed under the terms of the Creative Commons Attribution 4.0 International license.

### Patterns of bacterial interactions in GDM and normoglycemic groups.

**FIG 5:**
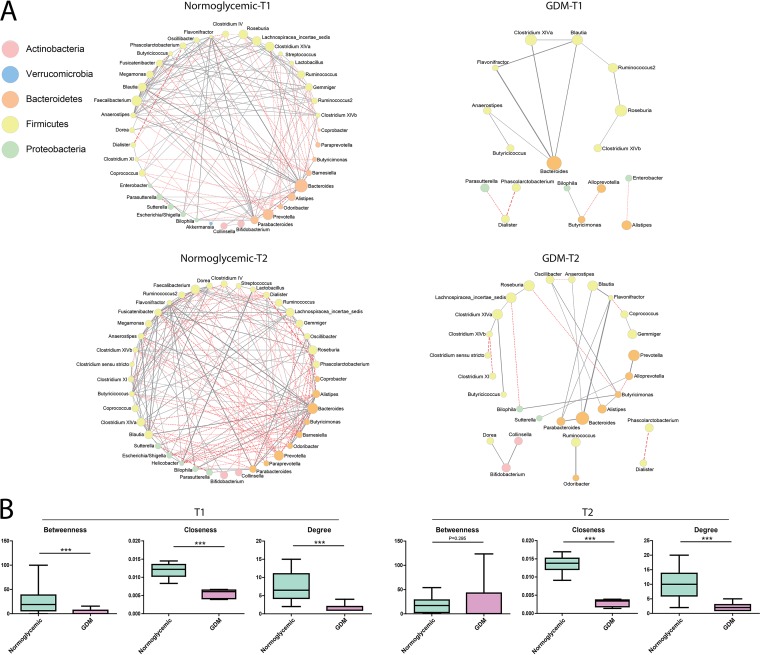
Patterns of bacterial interactions between women with GDM and control groups in T1 and T2 by cooccurrence network analysis (A) and statistical comparison of the topological variables (B). Lines between nodes represent the interbacterial correlations (edges), and gray solid lines and red dashed lines indicate positive and negative correlations, respectively. Green boxes represent women in the normoglycemic group; pink boxes represent women in the GDM group.

Cooccurrence networks were constructed to analyze the patterns of bacterial interactions (FDR < 0.1) among gut microbiota in the normoglycemic and GDM subjects in T1 and T2. Significant differences in the cooccurrence pattern were observed between the two groups in both T1 and T2 (Fig. 5). In T1, there were 152 associations or edges, including 60 negative ones, in the network of the control group, in contrast to 15 edges, including 4 negative ones, in that of the GDM group (Fig. 5A). In T2, the network contained 196 edges, including 91 negative ones, in the normoglycemic group and 28 edges, including 8 negative ones, in the GDM group. In addition to being smaller, the network of the GDM group was also fragmented. Of note, *Firmicutes* was the dominant phylum in all of these networks, followed by *Bacteroidetes*, which mirrored the taxonomic composition of the study cohort (Fig. S2). Given the apparent differences between the GDM and control groups, we analyzed the microbial network structure using parameters of betweenness centrality (the number of shortest paths going through a node), closeness centrality (the number of steps required to access all other nodes from a given node), and degree (the number of adjacent edges) (22). Our results demonstrated significantly lower betweenness (*P* < 0.001; Wilcoxon signed-rank test), closeness (*P* < 0.001), and degree (*P* < 0.001) of the microbial network in the GDM group than in the control group in T1 (Fig. 5B). In T2, the significantly lower closeness (*P* < 0.001) and degree (*P* < 0.001) of the GDM group were maintained, although its betweenness was comparable with that of the control group.

## DISCUSSION

To our knowledge, this is the first study to have demonstrated that women who develop GDM in late gestation exhibit distinct dynamics of gut microbiota from early to midgestation. Differences in taxon composition between the GDM and control groups were identified in both T1 and T2. In addition, women who developed GDM exhibited a marked reduction in the dynamic changes of gut microbiota from T1 to T2. There were also prominent differences in interbacterial interactions between GDM and normoglycemic women. We did not detect any significant association between microbial taxa and multiple GDM-correlated glucolipid measures.

Consistent with a previous study (23), our data demonstrated significant changes in microbiota from early to midgestation in normoglycemic subjects. Compared with T1, there were elevations in the F/B ratios, increased proportions of 8 genera, and decreased proportions of 11 genera, as well as 20 discriminatory balances in T2. Some of these trends, which are consistent with previous observations (12), appeared to be part of extensive gestational alterations in gut microflora during pregnancy. Healthy adults typically exhibit overall stability in the enteric microbial community and share major features, such as domination of *Bacteroidetes* and *Firmicutes* together with minor constituents such as *Actinobacteria*, *Proteobacteria*, and *Verrucomicrobia*, despite wide interpersonal variations in environmental, socioeconomic, and dietary aspects (24). Similar changes have been observed between microbiotas across various diseases (24). Intriguingly, as pregnancy progresses, the taxonomic changes of gut microbiota resemble those found in men and nonpregnant women that are associated with inflammation and fat deposition (12). In contrast to the broad changes in gut microbiota that occur during normal pregnancy, there were clearly fewer taxonomic and functional shifts and smaller discriminatory balance changes in gut microbiota from early pregnancy to midpregnancy in women who later developed GDM. We postulate that the lack of normal dynamic changes or static gut flora may contribute to the pathogenesis of GDM. Importantly, we used the balance tree approach to take into account the interactions between taxa by evaluating associations between GDM and sets of microbes rather than individual taxonomic units (24). The results from balance tree analysis and LDA were consistent in showing the apparent reduction in inter-time point variability of gut microbiota in GDM-developing women. In addition, results from balance tree analysis revealed differential variations of individual OTUs within a genus, illustrating the importance of subgenus taxa dynamics in GDM pathogenesis.

Earlier studies showed that inflammation, insulin resistance, and impaired glucose tolerance are positively associated with *Blautia* (14, 25), *Rothia* (26), and *Bilophila* (27) and negatively associated with *Bacteroides*, *Parabacteroides* (28), and Acinetobacter (29). In our study, we found that *Blautia*, *Rothia*, and *Bilophila* were less abundant whereas *Bacteroides*, *Parabacteroides* (28), and Acinetobacter were more abundant in T1 than in T2 in normoglycemic women, which is consistent with the increase in insulin resistance seen as pregnancy progressed. Interestingly, we found no differences in the abundances of these microbes from T1 to T2 in GDM women, suggesting that these gut flora may contribute to enhanced insulin resistance in early pregnancy in this group. Together, these findings suggest that dysbiosis, or an imbalance of gut microflora, may contribute to insulin resistance and glucose intolerance later in pregnancy.

Associations between gut microbes and GDM have been observed in previous studies (14, 30). Crusell et al. identified significant differences in several genera, including *Collinsella*, *Blautia*, and *Rothia*, between GDM and control groups during the third trimester of pregnancy and postpartum (14). However, since that study was conducted in late pregnancy, it is unclear whether these microbes cause GDM. In the present study, we identified a large multitaxon shift between GDM and the control group in both T1 and T2, indicating that some microbiota alterations from early pregnancy to midpregnancy were associated with later development of GDM.

Recently, bacteria that produce short-chain fatty acids (SCFAs) have been implicated in the pathogenesis of metabolic diseases, in particular, type 2 diabetes (31, 32). Our study provided multiple lines of evidence linking SCFA-producing bacteria with development of insulin resistance and GDM. Specifically, we found decreased relative abundances of the butyrate-producing bacterium *Coprococcus* (33) and the lactate-producing bacterium *Streptococcus* (both are genera known to contain multiple SCFA-producing species) in both T1 and T2 in GDM women. Findings from balance tree analysis also confirmed similar shifts in *Coprococcus* and *Streptococcus* associated with GDM in both T1 and T2 (see Fig. S5 in the supplemental material; see also Table S4 in the supplemental material). These results suggest that levels of SCFA-producing microbes may be inversely related to the early development of glucose intolerance. Consistent with this possibility, microbial fermentation of dietary fiber produces SCFAs, which has been proven to be beneficial to many aspects of host metabolism. For example, SCFAs may activate G-protein-coupled receptors (GPRs), increase secretion of gut hormones (e.g., glucagon-like peptide 1 and peptide YY) by intestinal epithelial L cells and leptin by adipocytes, and suppress production of proinflammatory cytokines (34), thereby regulating insulin sensitivity (35) and the pathophysiological course of GDM (36).

In the current study, the positive associations between *Megasphaera*, *Eggerthella*, and GDM were consistent with a previous report (20). Some strains of Eggerthella lenta have been associated with imidazole propionate production and insulin resistance (6). The denominator taxa of *Bacteroides* identified by balance tree analysis, which were more abundant in GDM women in both T1 and T2, have been associated with serum proinflammatory interferon gamma levels (37), insulin resistance (38), and plasma glucose levels (39). In summary, our study demonstrated significant differences in the dynamics of gut microbiota from early to middle pregnancy between normoglycemic and GDM women. The reduced inter-time point variability in gut microbiota in women who develop GDM implies that dysbiosis in gut microbiota begins in their early pregnancy. This finding suggests that gut microbiota could potentially serve as a biomarker for early detection of GDM. Further studies are needed to establish the causal relationship between microbial community shifts and development of GDM and thereby identify potential therapeutic targets.

## MATERIALS AND METHODS

### Study population and design.

This was a nested case-control study that was conducted in Beijing Obstetrics and Gynecology Hospital from July 2017 to December 2018. Women at 18 to 45 years of age and with a singleton pregnancy were recruited at gestational week 8 to week 12 and followed monthly until a 75-g oral glucose tolerance test (OGTT) was performed between gestational weeks 24 and 28 to screen for GDM. Only participants with complete clinical information were included in the analysis. Subjects were excluded if they had had chronic medical conditions, including hypertension, type 2 diabetes mellitus, and heart or kidney diseases, or had reported use of antibiotics or medications that would affect gastric or intestinal microbiota within the 2 months prior to entry. The study was approved by the Ethics Committee of Beijing Obstetrics and Gynecology Hospital (2017-KY-015-01). Written informed consent was obtained from every participant. All procedures were performed in compliance with the Declaration of Helsinki.

### Clinical measurements.

Baseline anthropometric measurements were completed at recruitment using a standardized protocol. Clinical data were collected by medical record review. Prepregnancy body weight was self-reported.

GDM was diagnosed at gestation week 24 to week 28 according to American Diabetes Association (ADA) criteria, in which at least one of the following criteria need to be met during a 75-g OGTT: fasting plasma glucose (FPG), ≥5.1 mmol/liter; 1 h glucose, ≥10.0 mmol/liter; 2 h glucose, ≥8.5 mmol/liter (40).

### Sample collection and determination.

**(i) Blood sample measurement.** Venous blood samples were collected from participants following an overnight fast at 8–12 weeks and 24 to 28 weeks gestation. The serum glucose level and lipid panel were determined as described in a previous study (41).

**(ii) Fecal sample collection, DNA extraction, PCR amplification, and sequencing.** Fecal samples were collected at home by the participants using a PSP Spin Stool DNA Plus kit (Stratec Biomedical, Birkenfeld, Germany) following a standardized procedure, shipped immediately to the laboratory on dry ice, and stored at –80°C. Genomic DNA was extracted using a TIANamp Stool DNA kit (Tiangen Biotech, Beijing, China) according to the manufacturer’s protocols. The hypervariable V3-V4 region of the bacterial 16S rRNA genes was amplified using primers 341F (5′-CCTACGGGRSGCAGCAG-3′) and 806R (5′-GGACTACVVGGGTATCTAATC-3′). The PCR was performed in a 30-μl mixture containing 15 μl of 2× Kapa Library Amplification ReadyMix, 1 μl of each primer (10 μM), and 50 ng of template DNA. Amplicons were gel purified and quantified using Qubit 2.0 (Invitrogen, MA, USA). After preparation of the library, sequencing was performed on a MiSeq platform to generate paired-end reads of 250 bp (Illumina, CA, USA). DNA extraction, library construction, and sequencing were conducted at the Realbio Genomics Institute (Shanghai, China).

### Descriptive analysis of general characteristics.

General characteristics of the participants in the two groups were compared using Student's *t* test for analysis of continuous variables of normal distribution, the Wilcoxon signed-rank test for continuous variables of nonnormal distribution, and the chi-square test for categorical variables.

### Taxonomy classification and statistical analysis.

High-quality reads were clustered into OTUs using Usearch (v7.0.1090) in QIIME (v1.9.1; http://qiime.org/scripts/pick_otus.html) with a similarity threshold of 97%. Taxonomy was assigned to individual OTUs using the RDP classifier and RDP database of release 11 (http://rdp.cme.msu.edu/).All the samples were randomly subsampled to equal depths of 21,073 reads prior to the calculation of alpha and beta diversity metrics. Low-abundance taxa were removed if one of the following two criteria was met: (i) abundance below the relative abundance cutoff of 0.1%; (ii) presence in less than 50% of the samples in either the control or GDM group in T1 or T2. Alpha diversity was assessed using the Shannon diversity index and beta diversity by weighted UniFrac distance analysis and PCoA. Paired samples were used for inter-time point comparison between T1 and T2 within the normoglycemic or GDM group.

LDA, the Wilcoxon signed-rank test, and balance tree analysis were employed to analyze the microbiota shifts. FDR was calculated using the Benjamini and Hochberg method (p.adjust function in R). For the Wilcoxon signed-rank test, the abundance data were subjected to centered log ratio (CLR) transformation. The microbial functions were predicted with PICRUSt (42) using the 16S rRNA gene sequences, whereby functional differences were examined. Generalized linear models (GLMs) were used to explore the significantly different genera between two groups after controlling for possible confounding factors, including BMI, TC, and TG (see Table S1 and Table S2 in the supplemental material). Balance tree analysis (21) was performed to examine changes of taxa based on correlation clustering performed according to the user instructions (https://github.com/biocore/gneiss). A taxon was removed if it was present in fewer than 10 samples in each comparison.

10.1128/mSystems.00109-20.7 TABLE S1 GLM analysis of different genera with controlling variables (*P* < 0.05). Download Table S1, XLSX file, 0.01 MB. Copyright © 2020 Zheng et al. 2020 Zheng et al. This content is distributed under the terms of the Creative Commons Attribution 4.0 International license.

10.1128/mSystems.00109-20.8 TABLE S2 GLM analysis of independent associations of BMI/TC/TG with GDM development. Download Table S2, XLSX file, 0.01 MB. Copyright © 2020 Zheng et al. 2020 Zheng et al. This content is distributed under the terms of the Creative Commons Attribution 4.0 International license.

10.1128/mSystems.00109-20.9 TABLE S3 List of discriminatory branches based on balance tree analysis (FDR 0.1). Download Table S3, XLSX file, 0.01 MB. Copyright © 2020 Zheng et al. 2020 Zheng et al. This content is distributed under the terms of the Creative Commons Attribution 4.0 International license.

10.1128/mSystems.00109-20.10 TABLE S4 Taxon list of discriminatory branches based on balance tree analysis (FDR 0.1). Download Table S4, XLSX file, 0.3 MB. Copyright © 2020 Zheng et al. 2020 Zheng et al. This content is distributed under the terms of the Creative Commons Attribution 4.0 International license.

Cooccurrence networks were constructed to investigate the patterns of bacterial interactions in the GDM and control groups. For Spearman analysis, the relative abundances of taxa were subjected to CLR transformation and then the Spearman correlation coefficients between genera were computed using R. Statistically significant associations (FDR < 0.1) were used to construct the cooccurrence networks, which were visualized using Cytoscape (43).

Spearman correlation coefficients were also calculated to investigate the association between individual bacterial genera and measures of glucolipid metabolism, including glycemic parameters and lipid profiles.

### Data availability.

Genomic data are fully accessible at the Sequence Read Archive (SRA; https://www.ncbi.nlm.nih.gov/sra) under BioProject accession number PRJNA556764.

## Note Added after Publication

In Table 1, fourth row, first column, “Primipara” was changed to “Multipara.” In the “Gravidity” row, second and third columns, 17 (54.84) and 51 (48.54) were changed to 14 (45.16) and 53 (51.46), respectively.
