# Supplementary figures and images for: Gestational Diabetes Mellitus Is Associated with Reduced Dynamics of Gut Microbiota during the First Half of Pregnancy
Source: mSystems. 2020 Mar 24;5(2):e00109-20. doi: 10.1128/mSystems.00109-20 (PMC7093821; doi:10.1128/mSystems.00109-20)

Figure S1

A

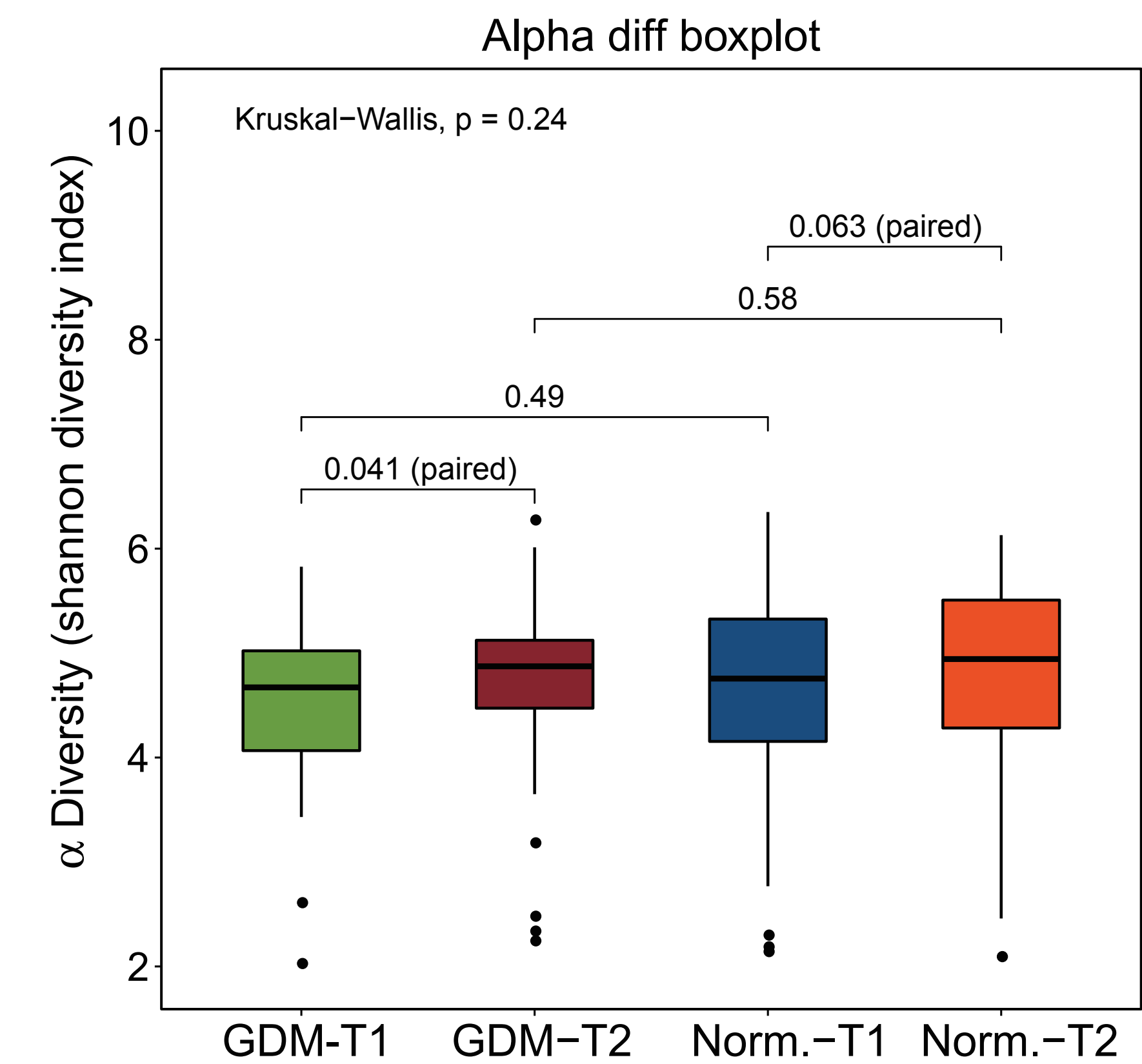

B

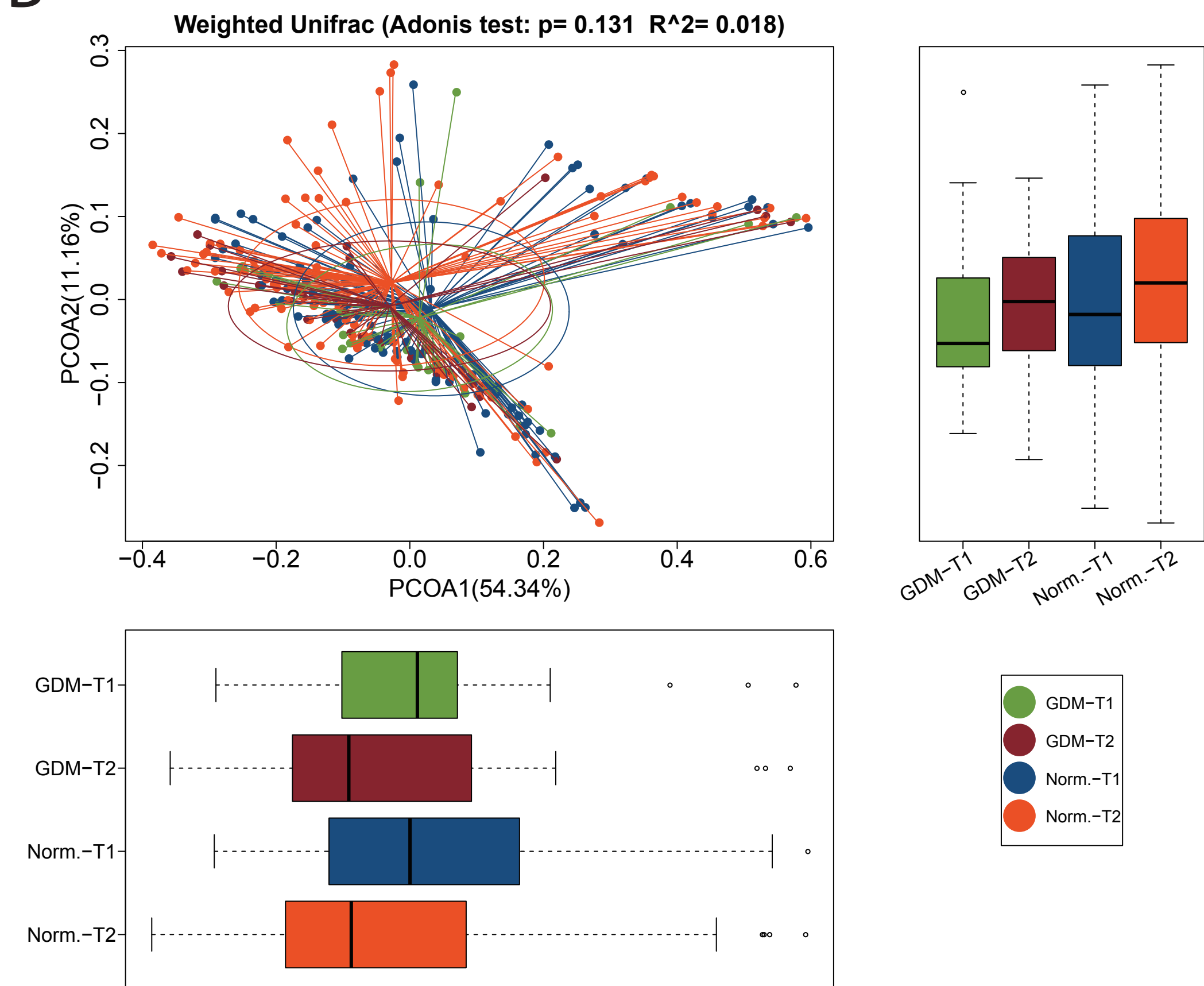

C

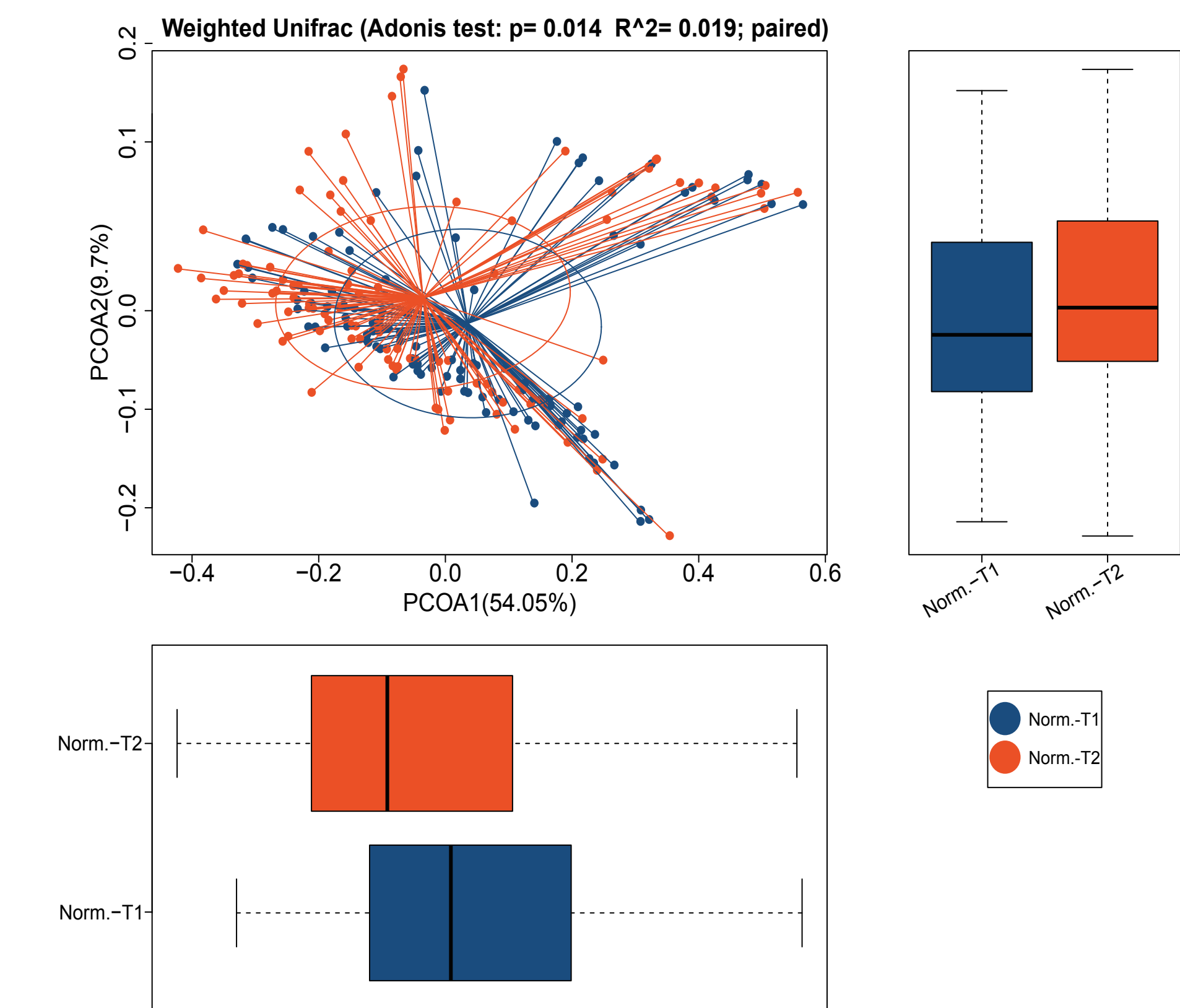

D

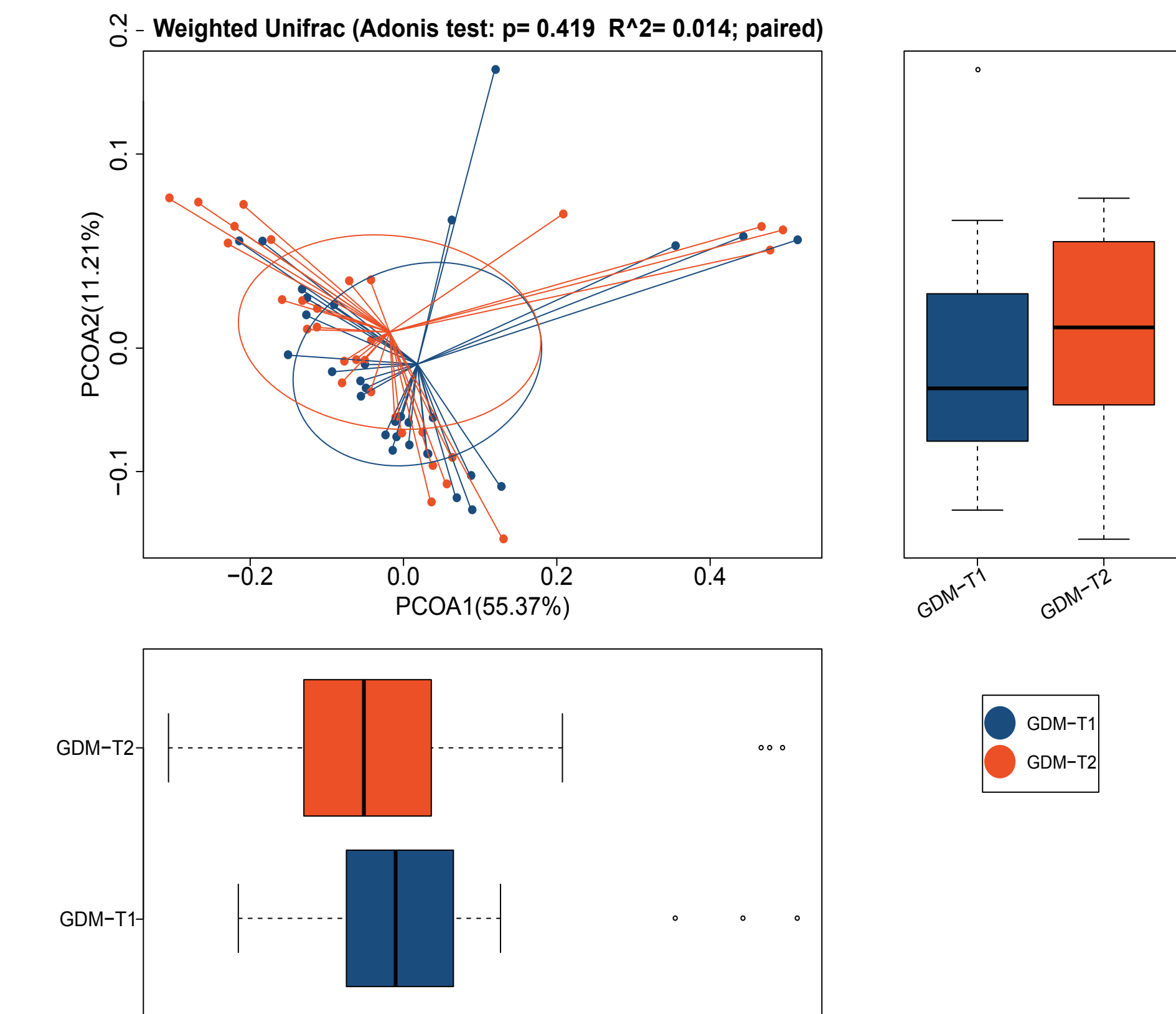

Supplement: FIG S1 [file mSystems.00109-20-sf001.pdf]

Figure S2

A

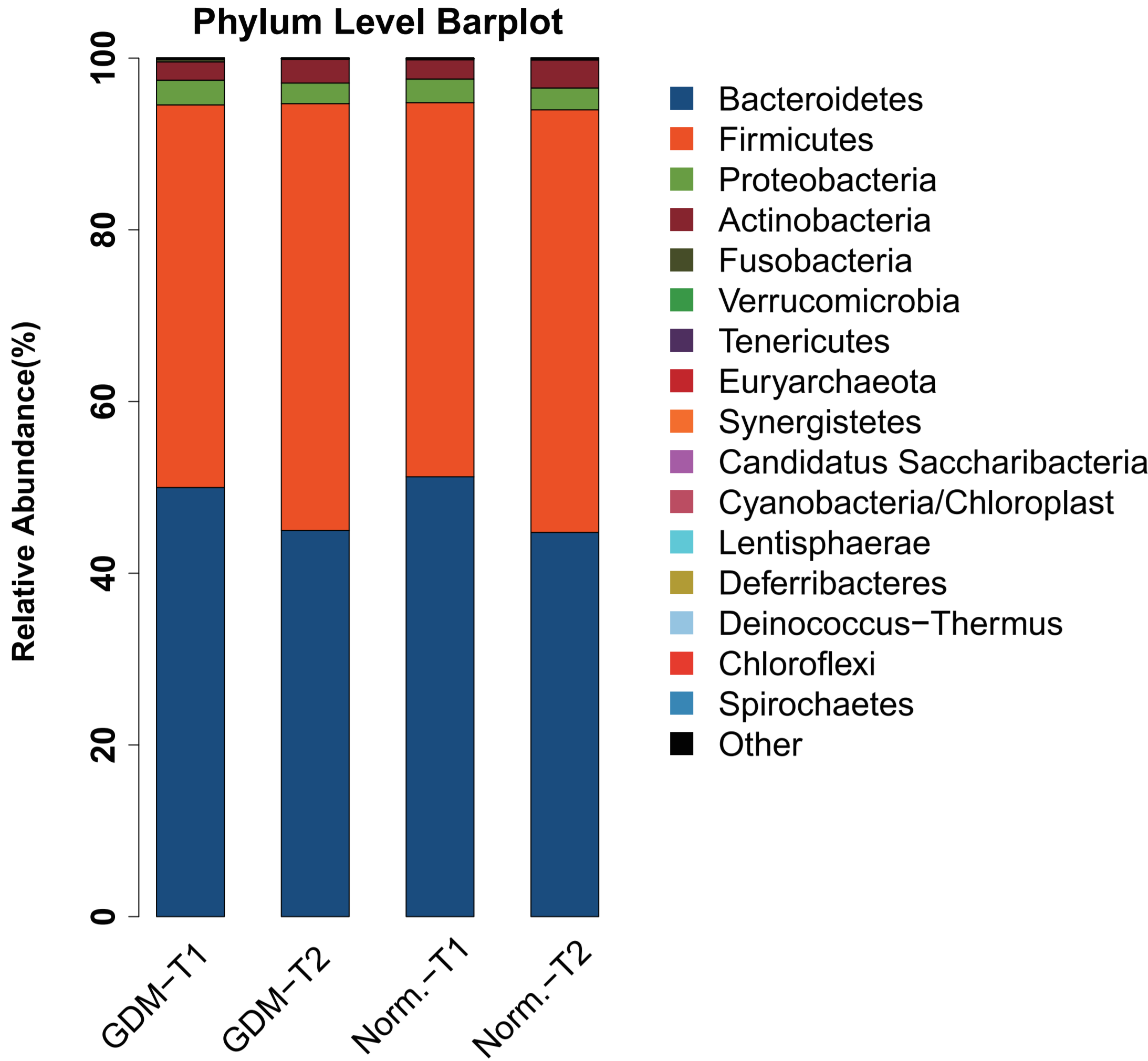

B

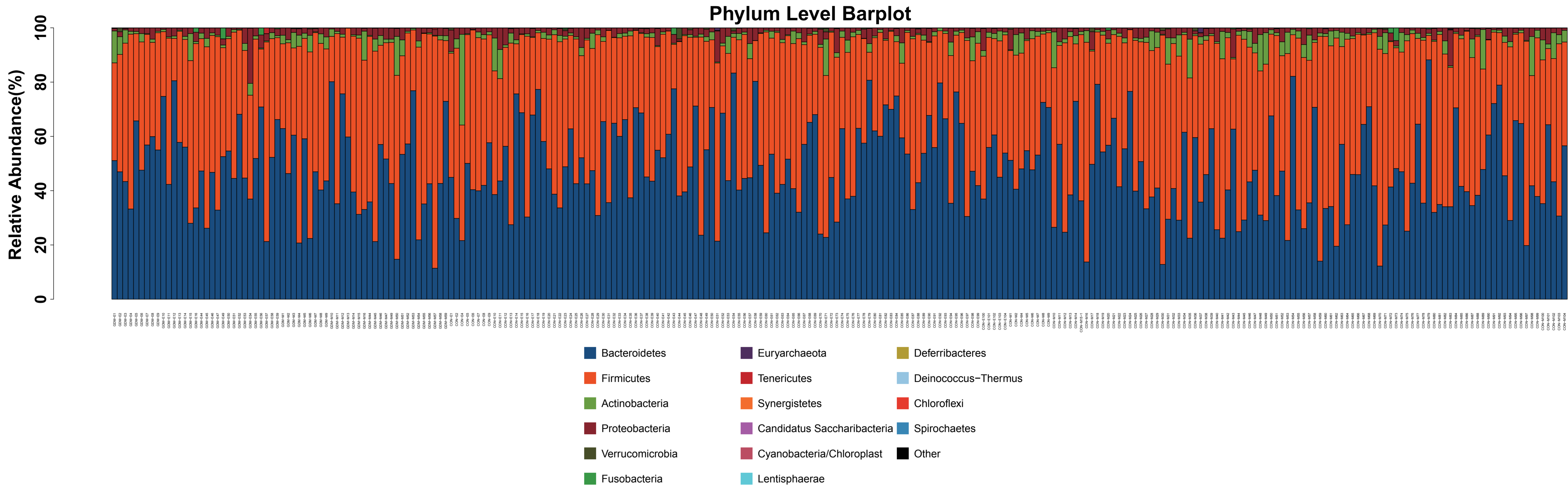

C

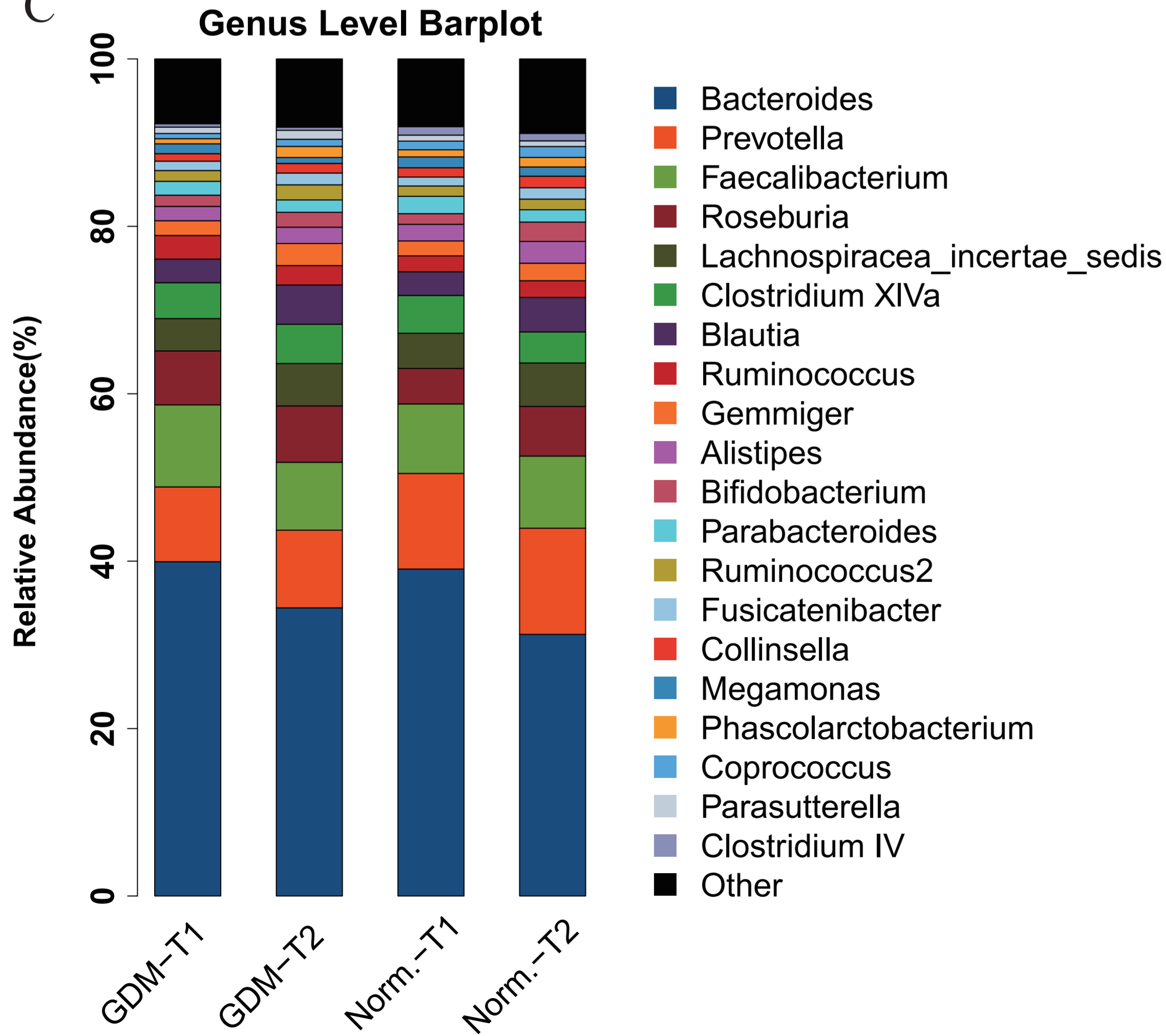

D

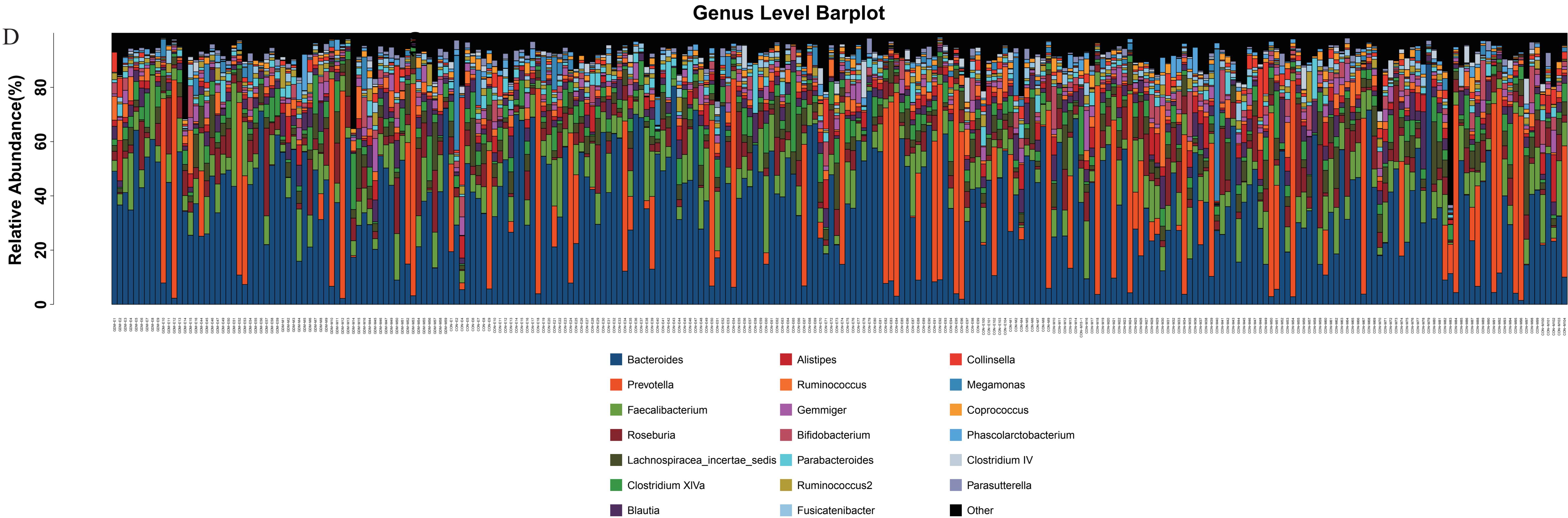

Supplement: FIG S2 [file mSystems.00109-20-sf002.pdf]

# Figure S3

A

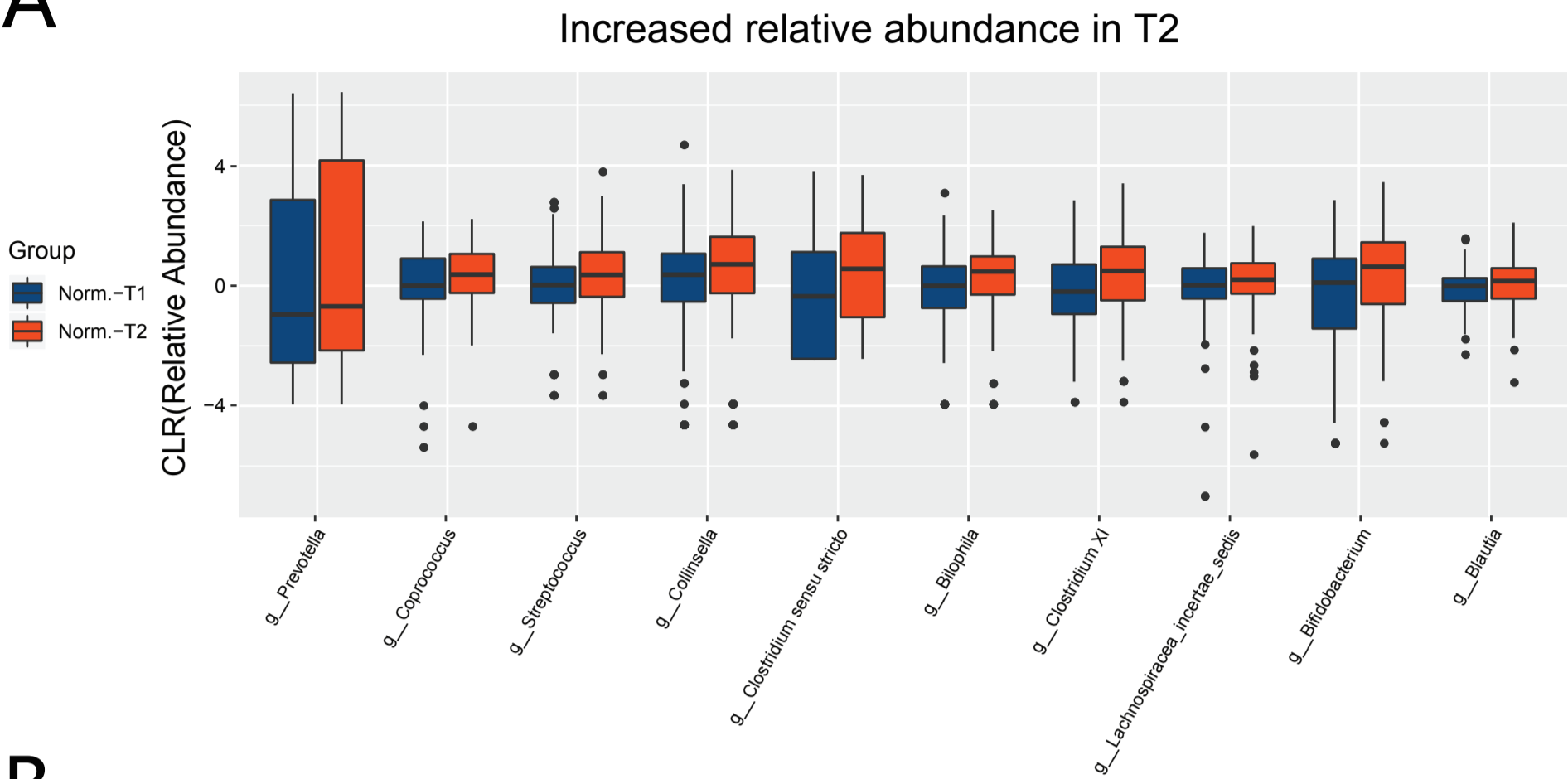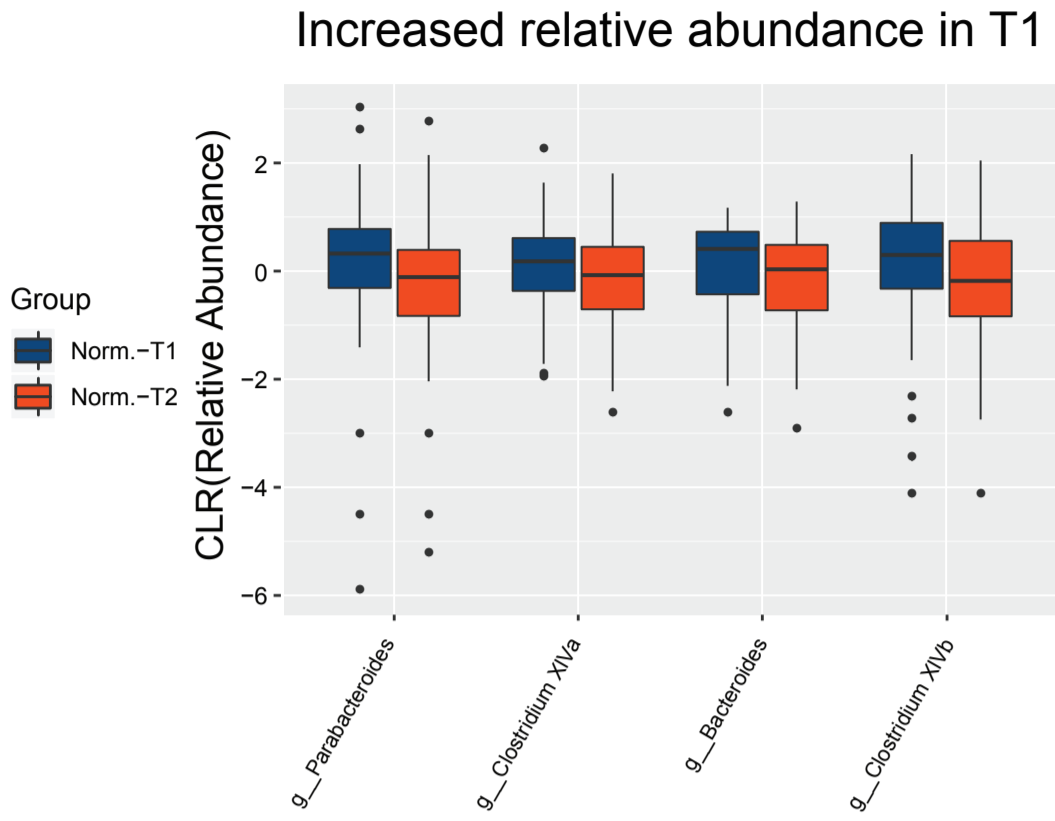

B

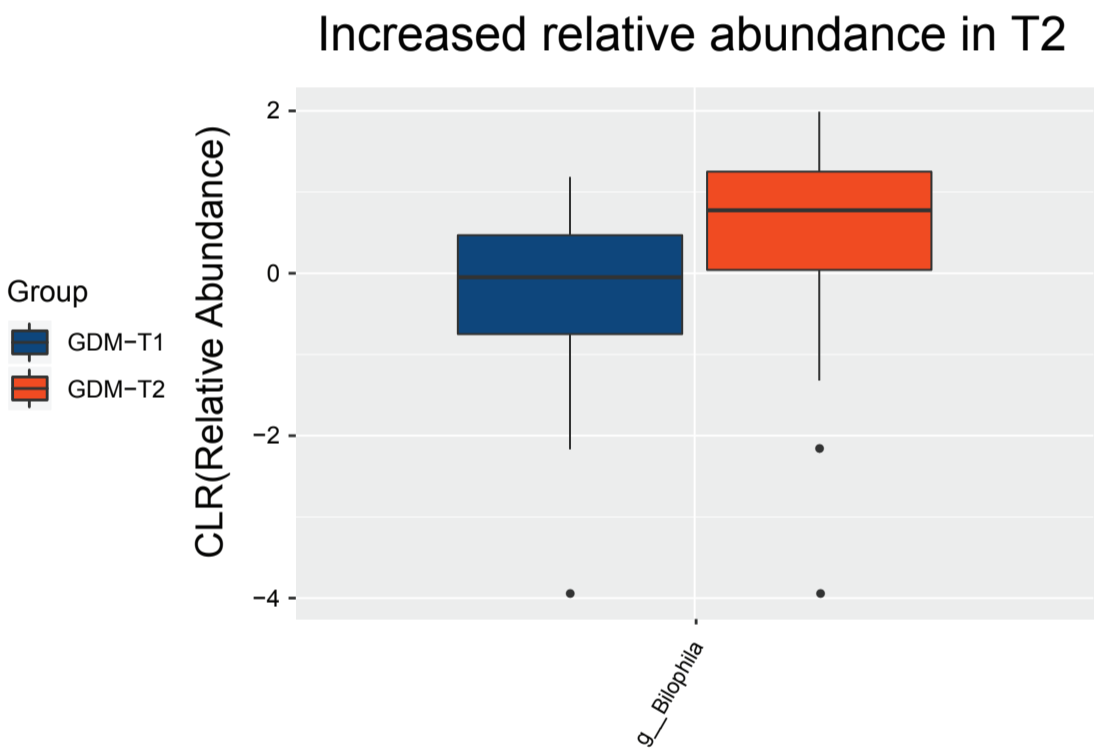

C

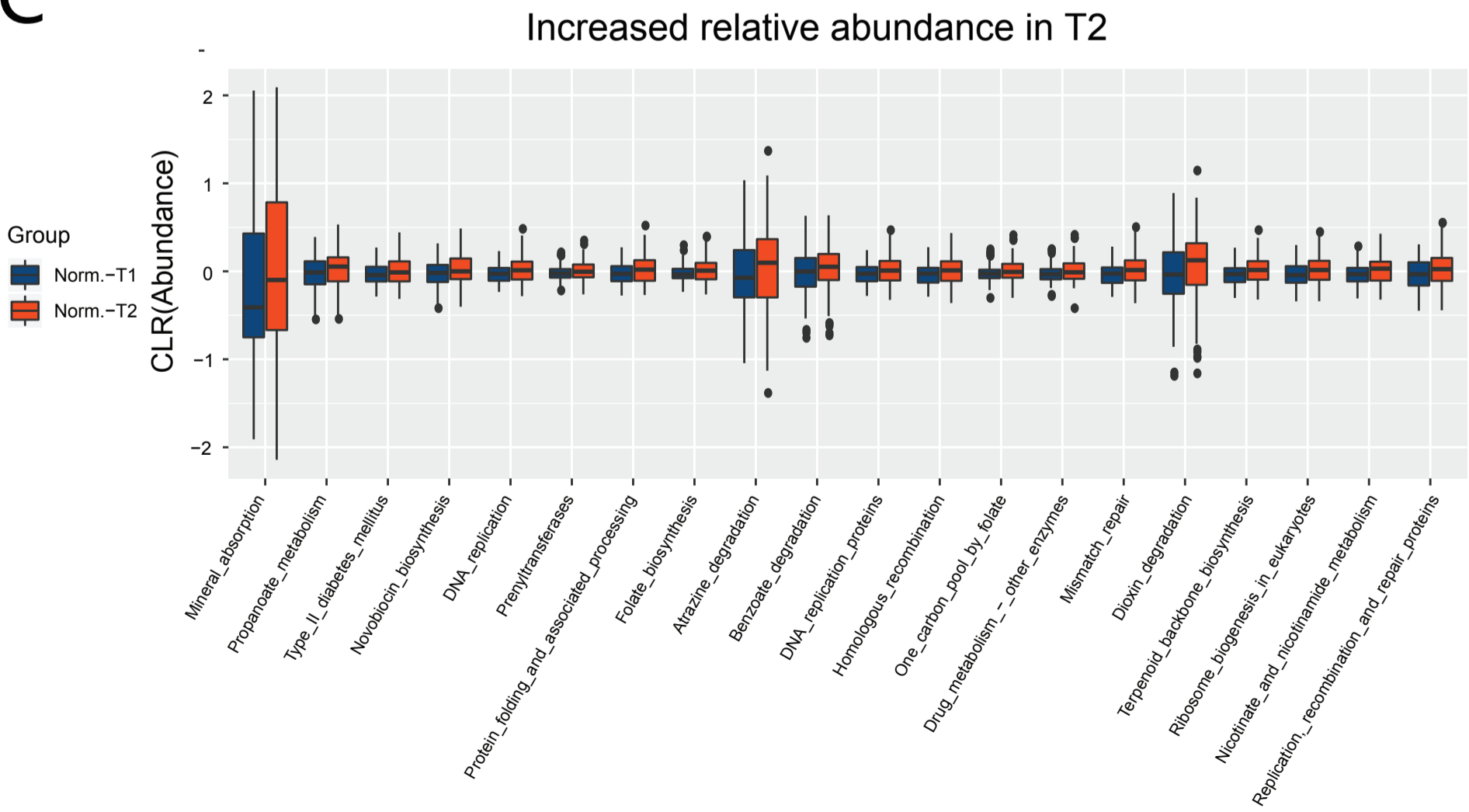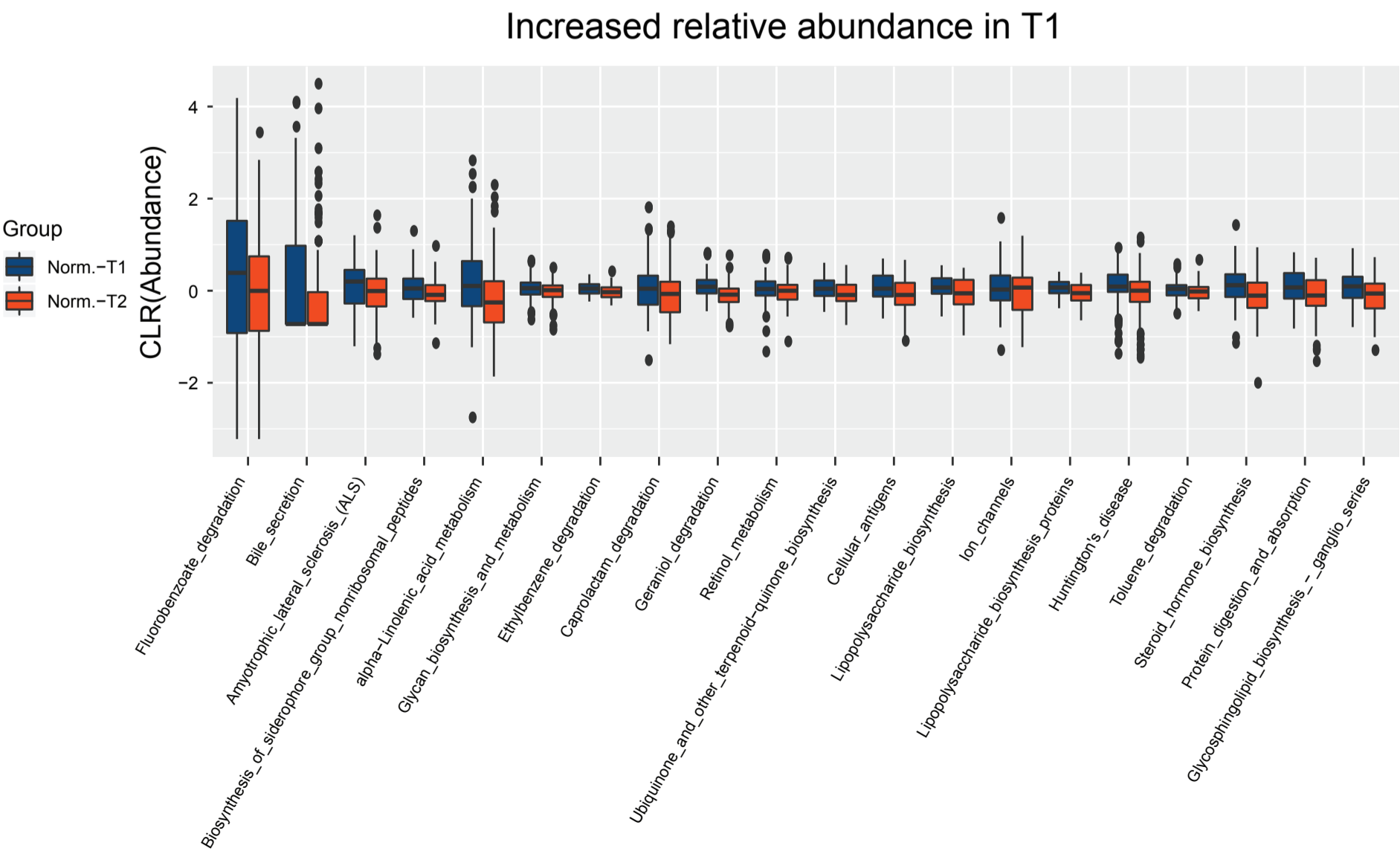

Supplement: FIG S3 [file mSystems.00109-20-sf003.pdf]

Figure S4

A

Normoglycemic (T1 vs.T2)

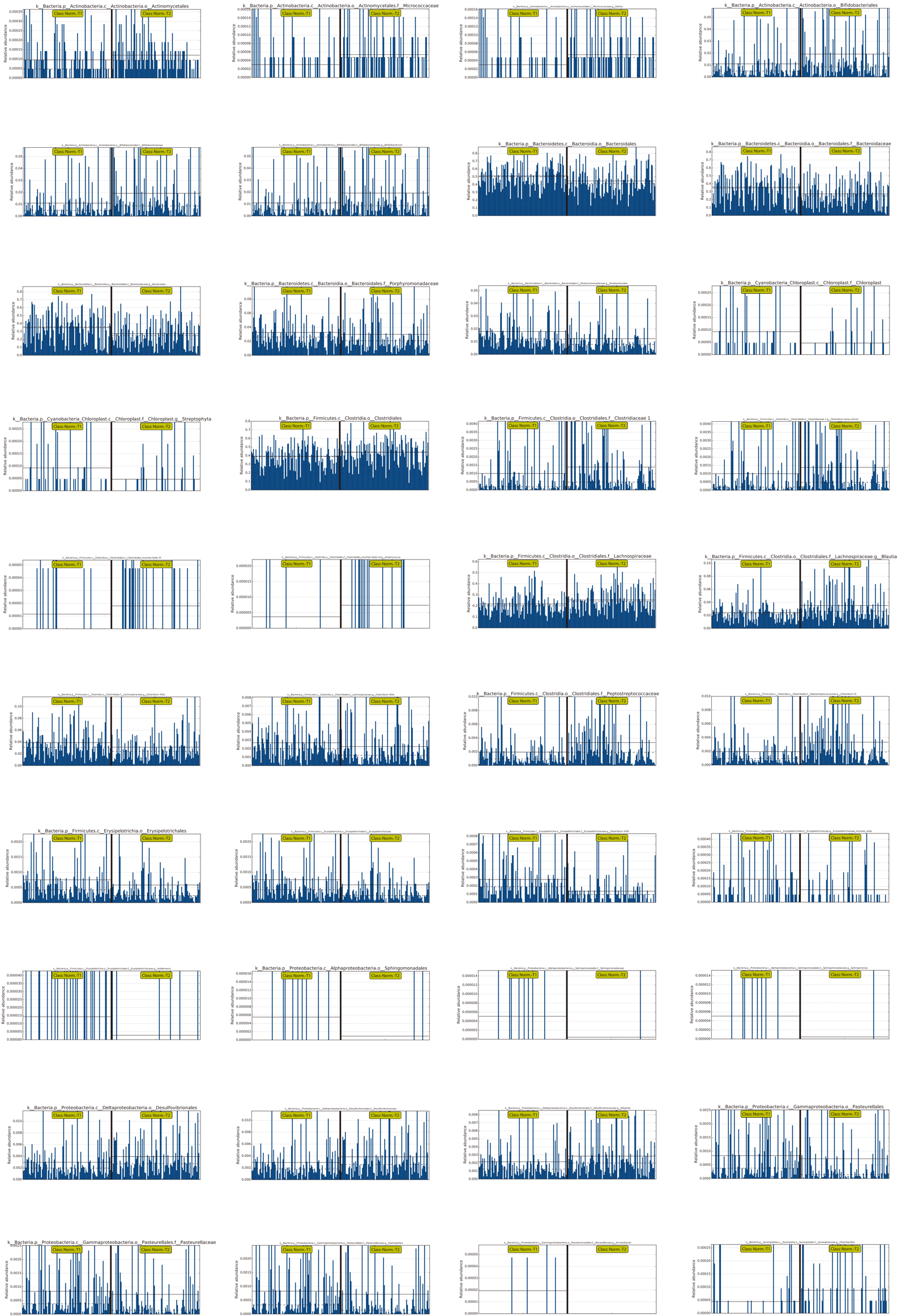

B

GDM (T1 vs.T2)

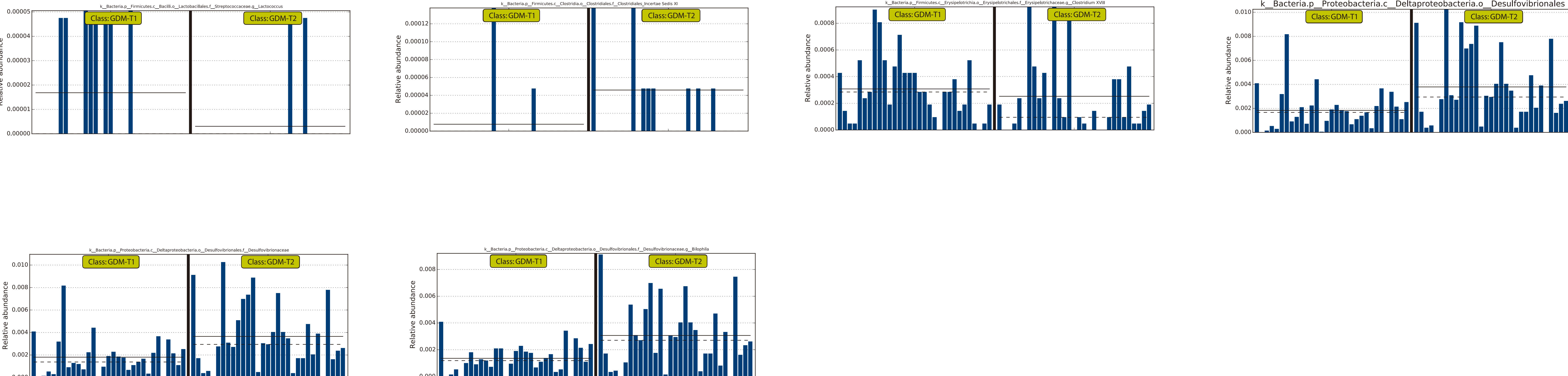

Supplement: FIG S4 [file mSystems.00109-20-sf004.pdf]

Figure S5

Normoglycemic vs. GDM (T1)

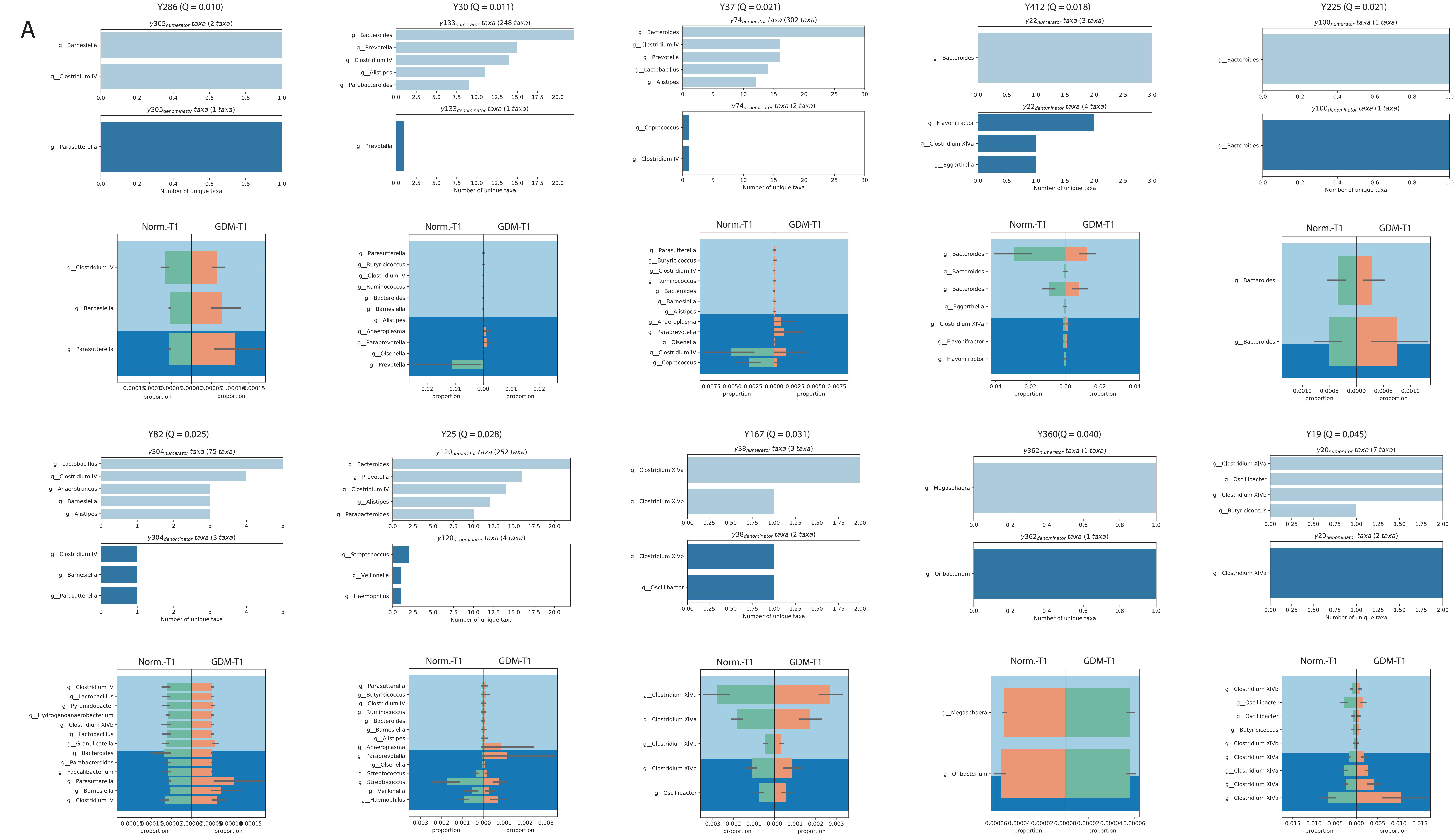

Normoglycemic vs. GDM (T2)

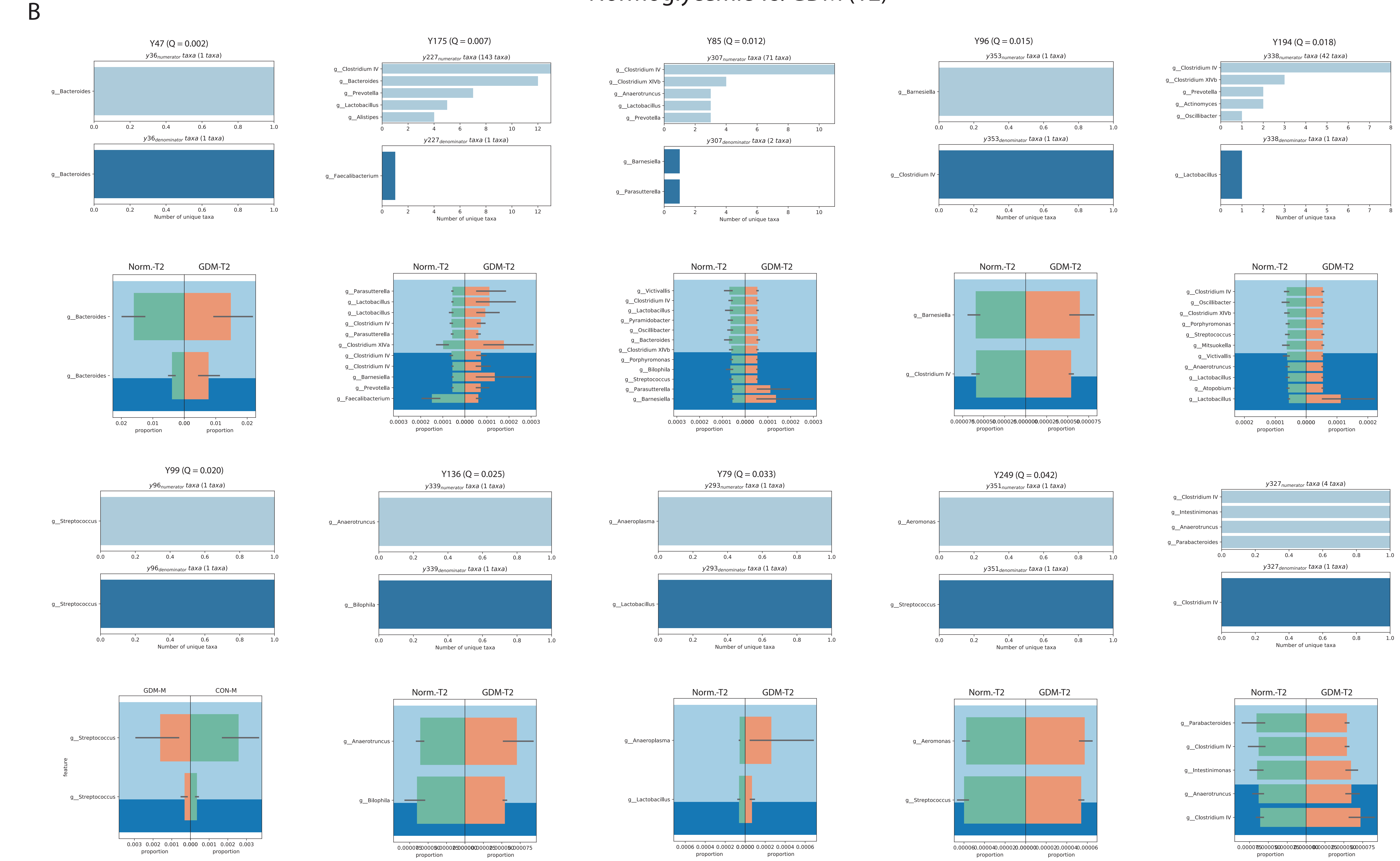

Supplement: FIG S5 [file mSystems.00109-20-sf005.pdf]

# Figure S6

## Normoglycemic (T1 vs.T2)

A

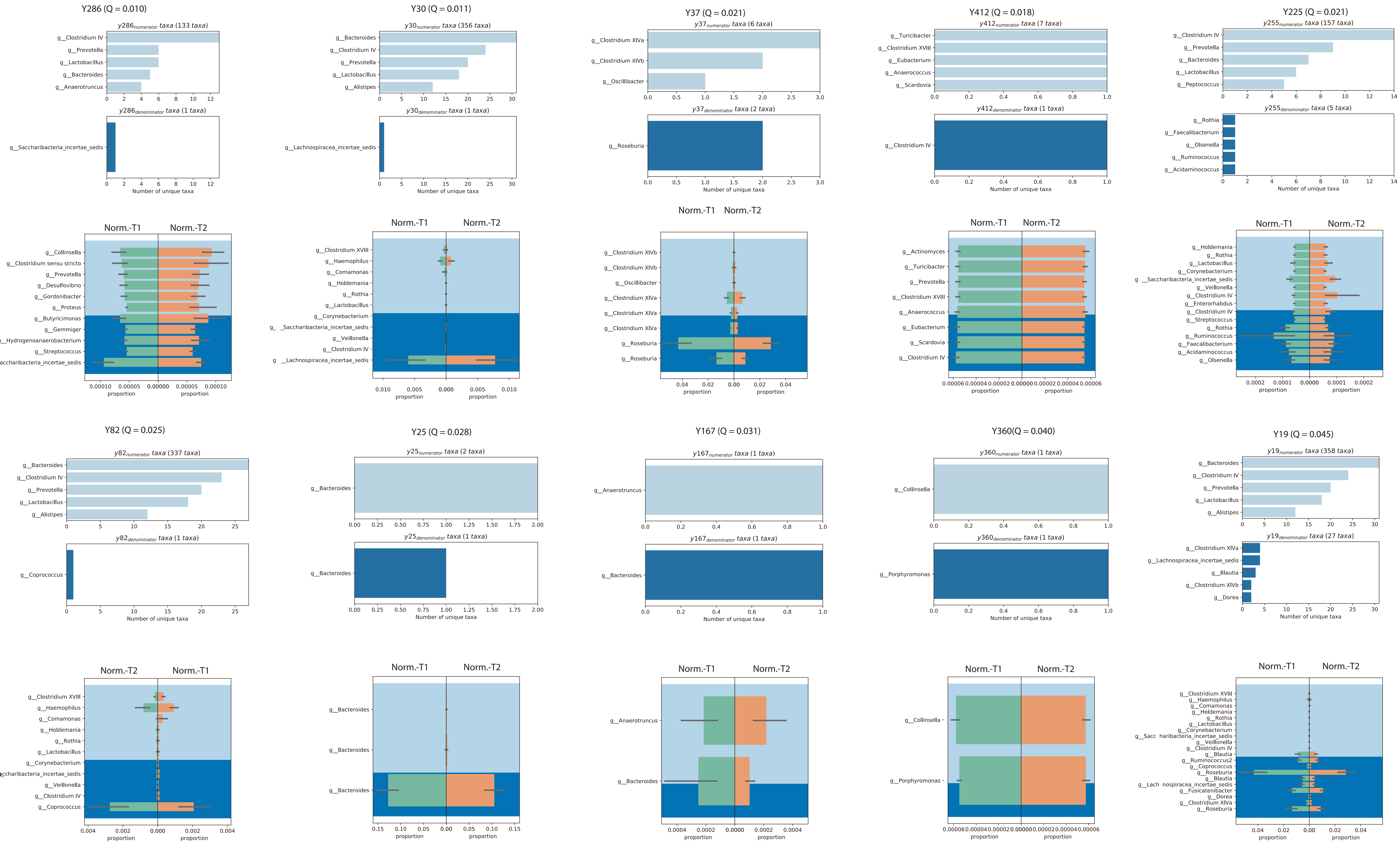

B

## GDM (T1 vs.T2)

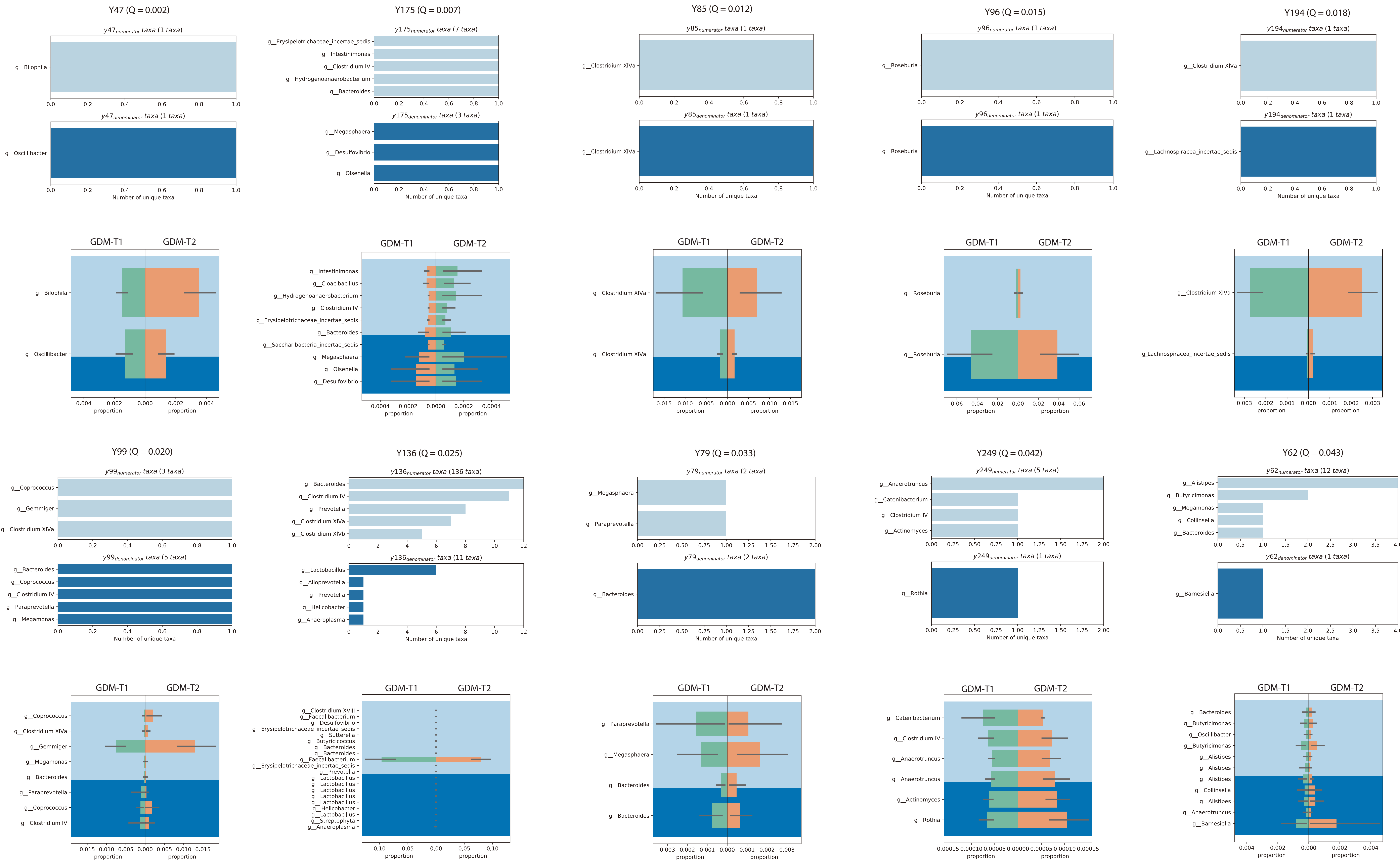

Supplement: FIG S6 [file mSystems.00109-20-sf006.pdf]
